# Feasibility and Acceptability of a Smartphone-Delivered Mindfulness Intervention for Stress Reduction in Adult Singaporeans: Pilot Randomized Controlled Trial

**DOI:** 10.2196/77793

**Published:** 2025-08-19

**Authors:** Alessandro Sparacio, Jonathan Nicholas Davies, Erin Lee, Jeroen Antonius Johannes Schmitt

**Affiliations:** 1 Human Development Institute for Human Development and Potential (IHDP) Agency for Science, Technology and Research Singapore Singapore; 2 School of Psychology The University of Queensland St Lucia Australia; 3 Mindful Moments Singapore Singapore Singapore; 4 Singapore Institute for Food and Biotechnology Innovation (SIFBI) Agency for Science, Technology and Research Singapore Singapore; 5 Human Potential Translational Research Unit Yong Loo Lin School of Medicine National University of Singapore Singapore Singapore

**Keywords:** mindfulness, stress management, mobile health, mHealth, heart rate variability, HRV, randomized controlled trial, RCT, feasibility study, digital health, sham control

## Abstract

**Background:**

Fully decentralized self-administered mindfulness interventions show promise for stress reduction, but rigorous evaluations of their feasibility, acceptability, and effectiveness using both self-report and physiological measures remain limited. In Singapore, where mental health concerns rank as the top health care priority (46%), ahead of cancer (38%) and stress-related issues (35%), accessible and scalable interventions are urgently needed to address the significant economic burden of mental health conditions.

**Objective:**

This study aimed to evaluate a decentralized, 3-day self-administered mindfulness intervention with minimal supervision, compared with a sham control in Singaporean adults, examining effects on self-reported and physiologically measured stress responses. The inclusion of a sham control condition was intended to disentangle mindfulness-specific effects from demand characteristics and expectation effects, addressing a critical methodological gap in digital mindfulness research.

**Methods:**

This was a purely smartphone-based, decentralized pilot trial with no face-to-face components. Participants were recruited using web-based methods and, after providing informed consent, 60 adults were randomized to either a mindfulness intervention or a structurally matched sham control. The daily 10-minute mindfulness sessions guided participants to focus on present-moment breath and body sensations, while the sham control omitted a focus on present-moment awareness. Outcomes were assessed remotely, using self-report questionnaires (State-Trait Anxiety Inventory-6) and physiological data from smartphone-based photoplethysmography (heart rate variability; HRV). The study incorporated three methodological innovations: (1) a structurally equivalent sham control to match expectancy and credibility, (2) remote collection of HRV as an objective physiological biomarker, and (3) full decentralization allowing unsupervised multiplatform delivery. Feasibility was evaluated through recruitment, retention rates, and data quality. Acceptability was assessed through quantitative ratings and qualitative feedback.

**Results:**

The study demonstrated excellent feasibility, with near-perfect retention (59/60, 98.3%) and moderate HRV data quality (231/331, 69.8% valid signals). Acceptability ratings were high (mean 4.17, SD 0.53), with comfort/engagement receiving the highest scores (mean 4.27, SD 0.57) on a 5-point scale, exceeding established usability benchmarks for digital health interventions. Qualitative feedback identified technical challenges (HRV instability and device overheating) and scheduling difficulties. While Bayesian analyses did not detect significant group differences in stress reduction (Bayes factor_10_ [BF_10_]=0.03) or HRV improvement (BF_10_=0.2), both groups showed significant stress reductions (BF_10_=3.01×10^6^), suggesting that observed benefits may stem from nonspecific factors common to both interventions.

**Conclusions:**

This study demonstrates (1) the feasibility of conducting fully decentralized mindfulness trials with multimodal assessment, (2) the value of a mixed methods acceptability evaluation, and (3) the identification of key technical and control condition refinements necessary for future trials. By addressing methodological limitations through improved control conditions and objective measures, this study provides a foundation for more rigorous investigation of mindfulness-specific effects.

**Trial Registration:**

ClinicalTrials.gov NCT06765889; https://clinicaltrials.gov/study/NCT06765889

## Introduction

### The Public Health Burden of Stress in Singapore

Stress exerts a pervasive influence on psychological and physiological well-being, contributing to conditions such as depression and anxiety [1-3]. In Singapore, mental health concerns rank as the top health care priority among residents (46%), ahead of cancer (38%) and stress-related issues (35%) [4]. The economic costs are also considerable: symptoms of depression and anxiety are estimated to account for a 2.9% loss of Singapore’s gross domestic product. Addressing this burden requires interventions that are not only effective but also scalable, accessible, and acceptable to diverse populations beyond traditional clinical settings.

### Methodological Challenges in Digital Mindfulness Research

Mindfulness—particularly in digital, self-administered formats—has emerged as a promising strategy for supporting mental health at scale, with a growing body of evidence linking it to reductions in stress, anxiety, and emotional dysregulation [5,6]. In particular, self-administered mindfulness (SAM) interventions—which remove the need for trained instructors and in-person delivery—offer practical advantages regarding reach, cost-effectiveness, and ecological validity [7]. However, concerns remain about the strength of the evidence for such interventions, particularly when delivered digitally and evaluated in uncontrolled or self-selected samples. Recent reviews point to challenges, including small effect sizes, publication bias, reliance on self-report outcomes, and insufficient control for nonspecific treatment effects, such as placebo and demand characteristics [8-11]

Demand characteristics and placebo effects are particularly salient in digital mental health studies, including mindfulness research. Participants may overreport improvements because of perceived researcher expectations [12] or inflated beliefs about the intervention’s efficacy [13,14], especially when control conditions lack structural and experiential equivalence [9]. A high-powered, preregistered, multisite study [6] addressed this concern by comparing SAM to an active control (story listening), but its control condition lacked key features of meditation itself that may be needed to effectively control for nonspecific factors and fully isolate mindfulness-specific effects.

### Bridging Gaps in Decentralized Mindfulness Research

This study addresses these methodological limitations by pilot-testing and evaluating a fully decentralized SAM intervention, compared with a structurally matched sham control. This study aimed to assess the feasibility and acceptability of delivering both interventions entirely remotely, while exploring preliminary effects on stress-related outcomes. The design incorporated three methodological innovations intended to improve future scalability and internal validity: (1) a sham intervention designed to match the SAM intervention in structure, expectancy, and credibility, and capable of disentangling potential mindfulness-specific effects from nonspecific effects (eg, expectancy and demand characteristics); (2) remote collection of heart rate variability (HRV), an objective physiological biomarker of stress; and (3) full decentralization of all procedures via smartphone, allowing unsupervised delivery across multiple platforms to maximize reach and minimize barriers to participation and potential sources of bias.

### Study Aims and Hypotheses

Feasibility and acceptability were evaluated to inform the design of future trials. Feasibility was assessed through recruitment and retention rates (documented via a CONSORT [Consolidated Standards of Reporting Trials] flow diagram), cross-platform delivery evaluation (Qualtrics, [Qualtrics], WhatsApp [Meta], and Camera HRV [ASMA BV]), adherence to protocols (including outcome measurement completeness), and physiological data quality. To contextualize our benchmarks, we drew on evidence from a meta-analysis of smartphone app trials for depressive symptoms [15], which reported an average 26.2% dropout rate (73.8% retention) across 18 randomized controlled trials, rising to 47.8% (52.2% retention) after adjusting for publication bias. Notably, studies incorporating human feedback and in-app mood monitoring demonstrated lower attrition. Aligning with these findings, our trial integrated WhatsApp for real-time human support and the app Camera HRV for physiological self-monitoring—strategies hypothesized to mitigate dropout. However, given the pilot nature of our study and the need to ensure rigor for subsequent efficacy trials, we adopted stricter feasibility criteria than typical app-based studies: recruitment of 60 participants, retention >80%, and ≥70% valid HRV or State-Trait Anxiety Inventory-6 (STAI-6) adherence. These targets exceed both the unadjusted and adjusted retention rates reported by Torous et al [14], reflecting our emphasis on optimizing engagement in a small-scale pilot. Acceptability was assessed via a 17-item usability questionnaire, open-ended feedback, and measures of credibility and expectancy.

While acceptability thresholds were not predefined, we interpreted results using benchmarks derived from System Usability Scale (SUS) literature [15,16]. Specifically, a retrospective study by Brooke [15] highlights that mean item scores ≥4 on 5-point Likert scales (eg, *strongly agree*) correspond to SUS scores >80, a threshold associated with “excellent” usability in digital tools. Lewis [16] further underscores that such scores, when coupled with low variability (SD ≤0.8), reflect strong consensus among users—a critical marker of acceptability in pilot interventions. These criteria align with mobile health studies that simplify SUS principles for feasibility-focused trials while retaining methodological rigor.

Our primary intervention hypothesis posited greater self-reported stress reduction in the mindfulness group, compared with the sham control group. The secondary objectives explored (1) HRV as a physiological stress marker, (2) ecologic momentary assessments (EMA)–derived transient states (eg, fatigue), and (3) moderation by baseline traits (ie, Five Facet Mindfulness Questionnaire [FFMQ], resilience, and neuroticism). Bayesian methods were used to estimate effect sizes and uncertainty, prioritizing practical interpretation over dichotomous significance testing. By aligning our feasibility framework with evidence-based strategies to reduce attrition (eg, the use of human engagement tools) and setting ambitious retention targets, this pilot bridges gaps identified in previous app trials and lays a robust foundation for fully powered efficacy studies.

## Methods

### Ethical Considerations

This study was conducted in accordance with the Declaration of Helsinki and approved by the Agency for Science, Technology and Research (A*STAR) Institutional Review Board on November 26, 2023 (reference 2024-102). All participants provided informed consent online via a survey administered on the Qualtrics platform. To protect anonymity, all study data were deidentified. Phone numbers were collected separately for operational purposes (ie, participant compensation, HRV data collection, and prevention of duplicate submissions) and were permanently deleted upon study completion. Participants were reimbursed via PayNow, receiving SGD $5 (US $3.88) for each completed daily assessment and an additional SGD $5 (US $3.88) bonus for completing all assessments, up to a maximum of SGD $20 (US $15.52).

### Recruitment and Eligibility

Participants were recruited via an online screening form that assessed eligibility based on several predefined criteria to ensure the safety of the participants and the integrity of the research. To qualify, individuals had to be aged at least 21 years, proficient in English, and willing to comply with all the study procedures. They must not have practiced meditation within the previous 6 months, used recreational drugs, or had uncorrected vision or hearing impairments. The participants were also required to have no history of mental illness, no diagnosed major neurological or psychiatric condition within the past 6 months, and no current or recent use (within the past week) of psychoactive medications, including antidepressants, anxiolytics, hypnotics, or stimulants. Given that the study procedures were conducted online, participants were also required to have access to the internet and sufficient computer literacy to complete the screening and study tasks. Finally, individuals with any current or prior affiliation with Agency for Science, Technology and Research (A*STAR) research teams were excluded.

Before proceeding, the participants were informed that the screening included questions designed to determine their eligibility and that honest responses were essential. The survey was configured to present each screening question individually and to terminate immediately if any exclusion criterion was met. This approach was adopted to reduce unnecessary exposure to sensitive questions and to maintain participant comfort and confidentiality.

Phone numbers were collected to prevent duplicate submissions and to facilitate compensation via PayNow, a Singapore-based instant payment system that allows direct bank transfers using mobile numbers. Duplicates were flagged by the study coordinator, and data from ineligible participants were immediately discarded.

The eligible participants received a Qualtrics link to review the study protocol and the consent form. Because of the study’s minimal risk (no medication or incidental findings), consent was obtained remotely without a witness. Participants who declined consent were excluded. Those who consented completed additional screening questions about neurological or psychiatric conditions and concomitant medication; failure to meet the criteria resulted in automatic termination.

### Materials

All study materials (ie, the meditation scripts, the analytic code, and the deidentified data) are hosted on Open Science Framework (OSF) [17], and the protocol was preregistered (ClinicalTrials.gov NCT06765889). The design followed a randomized, double-blind, 2-arm, parallel-group framework and was conducted remotely over 4 days (1 day for consent and 3 days for the study procedures). This was an entirely remote pilot study conducted through smartphones, with no in-person interactions. This trial is reported in accordance with the CONSORT 2010 statement [18] and its extension for digital interventions, CONSORT-EHEALTH (Consolidated Standards of Reporting Trials of Electronic and Mobile Health Applications and Online Telehealth) [19]. The CONSORT checklist can be found in Multimedia Appendix 1.

### Procedure

#### Overview

After providing consent, the participants were aggregated into cohorts of 20 and notified that the study would commence within 2 weeks. The cohorts were initiated weekly, with additional cohorts added as needed to reach the recruiting target of 60 participants. Following calculations by Whitehead et al [20] for continuous outcomes, the sample size (n=60) provided adequate precision to estimate feasibility parameters. The eligible participants received the following via a WhatsApp message from the study coordinator: (1) a Qualtrics survey link with a unique anonymous access code and (2) instructions for downloading and using the Camera HRV app [21], a third-party smartphone app that measures HRV via photoplethysmography (PPG) without requiring external hardware. The guidelines included (1) proper measurement protocols, (2) steps for submitting the recorded data as a .csv file, and (3) a free download code for the Camera HRV app.

The HRV data were collected via the app, and the participants submitted the files through WhatsApp to the study coordinator.

#### Beginning of Study Measurements

The participants who were eligible to start the study were instructed to move to a quiet environment for 35 minutes. They downloaded the Camera HRV app, conducted an initial test HRV measurement, and self-reported their root mean square of successive differences (RMSSD) between normal heartbeats values in the Qualtrics survey. They then completed demographic questions (ie, age, gender, ethnicity, type of device used for the experiment, the operating system, and the corresponding version) and the following scales:

- Neuroticism subscale of the International Personality Item Pool (International Personality Item Pool-20) [22]; this scale consists of 20 items assessing self-reported levels of neuroticism (α=0.95; 95% CI 0.93-0.96). Example items include “I often feel blue” and “I am filled with doubts about things,” rated on a 5-point scale ranging from 1 (very inaccurate) to 5 (very accurate).
- Resilience Scale 14 [23]; the Resilience Scale 14 is a brief version of the Resilience Scale, comprising 14 items rated on a 7-point Likert scale (α=0.92; 95% CI 0.90-0.95). Example items include “I usually manage one way or another” and “I am determined.”
- FFMQ-Short Form (FFMQ-SF) [24]; the FFMQ-SF is a 24-item measure assessing 5 facets of mindfulness (α=0.77; 95% CI 0.67-0.84). The 5 facets are observing (eg, “I pay attention to sounds, such as clocks ticking, birds chirping, or cars passing”); describing (eg, “I’m good at finding words to describe my feelings”); acting with awareness (eg, “I find myself doing things without paying attention”); nonjudging of inner experience (eg, “I criticize myself for having irrational or inappropriate emotions”); and nonreactivity to inner experience (eg, “I perceive my feelings and emotions without having to react to them”).

#### Pre- and Postintervention Assessments

The STAI-Short Form (STAI-6) scale [25], EMA [26], and HRV were measured twice daily—once before and once after listening to each audio track.

The STAI-6 is a validated short-form version of the 40-item STAI. It consists of 6 items measuring current anxiety symptoms “right now” using a 4-point Likert scale (α=0.88; 95% CI 0.86-0.90). Example items include “I feel calm” and “I am tense.”

In the EMA, participants rated their current state using a slider scale from 1 (not at all) to 100 (very much). The following 7 questions were used to assess various psychological and physical states:

1. “Right now, I feel mentally sharp”—to assess cognitive clarity and alertness.
2. “Right now, I feel fatigued”—to measure levels of fatigue and exhaustion.
3. “Right now, I feel stressed”—to gauge current stress levels.
4. “Right now, I feel nervous”—to assess feelings of nervousness and tension.
5. “Right now, I feel depressed”—to evaluate depressive symptoms.
6. “Right now, I am in a good mood”—to measure positive affect and emotional well-being.
7. “I slept well last night”—to assess subjective sleep quality, this last item was measured only once per day.

HRV was measured to assess parasympathetic activation using the PPG-based Camera HRV app [21]. PPG-derived HRV via smartphone cameras has been validated in multiple studies as a practical alternative to an electrocardiogram (ECG) under controlled conditions. For example, a validation study demonstrated perfect agreement between smartphone PPG and ECG for RMSSD, a key time-domain HRV metric linked to parasympathetic activity, particularly in resting-state measurements [27]. The Camera HRV app specifically has been benchmarked against research-grade devices. In comparative testing against the Polar H7 chest strap (a device previously benchmarked against ECG), Camera HRV demonstrated strong agreement for both heart rate and HRV metrics. Specifically, it achieved a correlation of *r*=0.97 for heart rate during rest and paced breathing conditions. For HRV parameters, the app showed a correlation of *r*=0.78 for RMSSD, with improved agreement (*r*=0.87 for pNN50) after application of its artifact correction algorithms [21].

The participants were instructed to place their index finger fully over the smartphone’s camera and flash, and to remain still for 1 minute to minimize motion artifacts. The app automatically flagged recordings as “poor” if signal quality fell below preset thresholds (eg, irregular pulse detection or incomplete coverage). The participants were instructed to take up to 2 additional attempts in this scenario.

#### End-of-Study Assessments

Usability and acceptability were assessed using a structured 17-item questionnaire grounded in established usability frameworks, including the SUS [16] and technology acceptance models [28]. The questionnaire evaluated key dimensions identified in digital health research [29], such as clarity of instructions, engagement, comfort, perceived efficacy, and overall satisfaction with the study procedures and tools (α=0.92; 95% CI 0.89-0.95). The questionnaire included 17 items rated on a 5-point Likert scale (1=*strongly disagree*, 5=*strongly agree*), measuring the following:

- Ease of access and navigation, for example, “The survey was easy to access and complete.”
- Clarity of instructions, for example, “The instructions for conducting the meditations were clear.”
- Comfort and engagement, eg, “The meditations were pleasant.”
- Technical usability, for example, “It was easy to download the app used to measure heart rate.”
- Study procedures, for example, “The study procedures were reasonable in terms of time commitment and effort.”

Open-ended questions (eg, challenges and time commitment) were used to expand on quantitative data, following recommendations for mixed methods usability assessments [30].

The Perceived Awareness of the Research Hypothesis (PARH) scale [31] is a 4-item quantitative self-report tool designed to assess the potential impact of demand characteristics in research settings. The participants responded to statements using a 7-point Likert-type scale (1=*strongly disagree*, 7=*strongly agree;* α=0.91; 95% CI 0.87-0.95). Example statements include “I was aware of the researchers’ objectives in this study,” or “I was uncertain about the researchers’ intentions in conducting this research.”

To assess the participants’ expectations regarding the intervention’s effectiveness, a single item adapted from the Credibility/Expectancy Questionnaire [32] was administered during the final phase of postintervention testing: “How effective do you believe the meditation training has been in alleviating stress? (0=*not at all successful*, 10=*extremely successful*).”

To evaluate credibility, the participants responded to a two-part question:

1. “If you were informed that you might have received either meditation training or control training, which type do you believe you received? (*meditation and control*).”
2. “How confident are you in your answer? (0=*not at all confident*, 10=*extremely confident*).”

### Intervention Development

To ensure that the mindfulness and sham interventions were grounded in best practices and optimized for a brief, decentralized format, we conducted a formative focus group with 5 certified international mindfulness-based stress reduction (MBSR) instructors before the pilot trial. The primary objective was to gather expert consensus on the core components, duration, and structure of a mindfulness protocol suitable for a multiday, self-administered study with a nonclinical population experiencing stress. The expert panel’s recommendations directly shaped the core parameters of the final study design. To ensure accessibility for beginners in a remote context, the instructors guided us to focus on foundational practices, leading to the selection of exercises centered on attentional stability through breath awareness and mindful meta-awareness of thoughts and sensations.

The panel also advised that a brief daily session of approximately 10 to 12 minutes would be optimal for maximizing engagement and adherence. On the basis of this, we implemented a 3-day “taster” protocol, which was considered an appropriate duration to robustly test the feasibility and acceptability of the intervention before scaling to a longer trial. The focus group also informed the design of the sham condition, which highlighted the challenge of creating a credible control. The experts suggested leveraging common misconceptions about meditation (eg, that it is purely a relaxation or thought-engagement exercise), which led to our final design emphasizing free-flowing thought and multitasking rather than present-moment awareness. The summary and transcript of the focus group can be found on the OSF page of the project [17].

### Randomization and Experimental Conditions

#### Overview

Participants were randomly assigned via Qualtrics to 1 of 2 experimental conditions: a mindfulness intervention or a sham meditation condition. The randomization process was implemented using the built-in Randomizer feature within the Qualtrics Survey Flow. This tool was configured to assign participants to the experimental conditions in a 1:1 ratio using the “evenly present elements” option, ensuring a balanced allocation across the duration of the study. Both conditions included audio-guided sessions recorded by the same certified MBSR instructor to maintain structural equivalence in delivery. The sessions were embedded within the Qualtrics survey and could not be skipped for 10 minutes (matching the time of the audio track).

#### Mindfulness Intervention

The mindfulness tracks were written and recorded by a certified MBSR instructor from Singapore with more than a decade of experience. The intervention included 3 unique guided practices, each designed to strengthen attentional stability and meta-awareness, which are key mechanisms associated with effective mindfulness training and stress regulation [33-35]. The sessions focused on the following elements:

1. Attentional stability; the participants were guided to anchor their focus on present-moment bodily experiences, such as breath sensations, physical posture, or environmental stimuli, to enhance their ability to sustain attention without distraction.

2. Mindful meta-awareness; the practice encouraged nonreactive and nonjudgmental observation of thoughts, emotions, and bodily sensations, fostering acceptance and self-regulation skills.

Each track emphasized present-moment awareness using different modalities:

1. On day 1, formal mindfulness practice (ie, body awareness meditation)—participants were encouraged to be attentive to bodily sensations, surrounding sounds, and breath, training them to maintain awareness despite distractions.
2. On day 2, informal mindfulness practice (ie, mindful brushing of teeth)—the participants were guided to integrate mindfulness into the act of brushing their teeth, approaching this habitual behavior with curiosity and full attentiveness. The practice encouraged awareness of sensations, movements, and breath, fostering a mindful presence.
3. On day 3, breath awareness practice—participants were guided to observe the natural rhythm of breathing without trying to change it, reinforcing nonjudgmental awareness.

#### Sham Meditation Condition

AS and JND developed the sham meditation condition, with JND contributing expertise in implementing such control conditions [8]. After finalizing the sham, the mindfulness instructor reviewed it to ensure that no core mindfulness components remained. Designed to structurally mirror the mindfulness intervention, the sham condition intentionally excluded key mindfulness mechanisms (eg, attentional stability and meta-awareness). The goal was to create an experience that matched the mindfulness group in duration, delivery format, and voice, thereby controlling for nonspecific factors, while guiding participants through exercises that emphasized multitasking and free-flowing thought, creating an experience that felt meditative to novices without delivering active mindfulness training [8]. Specifically, the sham condition was designed to differ from the mindfulness intervention in two fundamental ways: (1) a lack of attentional stability; unlike the mindfulness condition, the sham sessions did not provide an anchor for attention, and participants were encouraged to allow their thoughts to wander freely, shifting between different ideas and tasks; and (2) a lack of metacognitive guidance; instructions omitted any emphasis on nonjudgmental awareness, acceptance, or self-observation; rather, participants were prompted to engage with multiple thoughts at once, emphasizing cognitive agility and rapid task switching.

Each sham session included the following: on day 1, associative thinking (eg, encouraging free-association thinking)—participants were guided to let their minds jump between thoughts, focusing on productivity and efficiency rather than present-moment awareness; on day 2, routine task engagement (ie, brushing teeth while thinking about tasks)—participants were encouraged to perform everyday activities (eg, brushing teeth) while reflecting on unrelated tasks; this approach emphasized multitasking without cultivating mindful curiosity or awareness; on day 3, creative exploration (eg, picking a thought and expanding on it)—participants were instructed to select a thought and expand on it through imaginative thinking. This exercise promoted mental flexibility and creativity but did not involve sustained focus on present experiences.

The script of the active and control condition, along with the corresponding audio tracks, can be found on the OSF page of the project [17].

### Daily Procedure

The participants repeated the same procedure on days 1, 2, and 3:

1. Complete the preintervention self-report assessments (STAI-6, EMA, and HRV)
2. Listen to a different mindfulness or sham meditation track, depending on group allocation
3. Sit quietly for 3 minutes to minimize confounding variables in the subsequent HRV assessments
4. Complete the postintervention self-report assessments (ie, STAI-6, EMA, and HRV)

The participants were instructed to complete their daily sessions at approximately the same time each day to ensure consistency. They were informed that the sessions on days 1 and 3 would each take approximately 35 minutes, while the day 2 session would take around 25 minutes. On day 3, after the traditional postintervention assessments, the participants also completed the end-of-study assessments: the PARH scale, the expectancy and credibility of the intervention, and the usability and acceptability questionnaire. Finally, the participants were asked to send the .csv files generated by the Camera HRV app via WhatsApp to the study coordinator, which encompassed all the HRV measurements taken during the study. The participants were then thanked for their participation, debriefed, and provided compensation via PayNow.

### Statistical Analysis

#### Primary Outcomes: Feasibility, Acceptability, and STAI-6

We first evaluated feasibility using three key metrics: (1) recruitment yield (completed to enrolled ratio), (2) retention rates, and (3) protocol adherence (STAI-6 and HRV completion percentages). Acceptability was assessed via the 17-item usability questionnaire (analyzed descriptively) and qualitative coding of open-ended responses. We implemented a Bayesian latent growth curve model using the *brms* package [36] to examine the effects of the mindfulness intervention on self-reported stress (measured via the STAI-6). We specified a model that included fixed effects for the time point of the assessment (preintervention vs postintervention), the condition (mindfulness vs sham), and their interaction; a covariate for day to account for potential daily fluctuations; and random slopes for time point that varied by participant to account for individual differences in responsiveness to the intervention.

We used weakly informative priors: normal (0, 1) for the fixed effects, normal (0, 5) for the intercept, and Cauchy (0, 2) for the random effects SDs. For each model, we ran 4 Markov Chain Monte Carlo chains with 4000 iterations each, using an adaptation delta of 0.95 to ensure adequate chain mixing and convergence. To evaluate the evidence for the experimental manipulation, we compared the full model (including the time point × condition interaction) with a null model without this interaction term. We used a Bayes factor (Bayes factor_10_ [BF_10_]) comparison between the full and null models to quantify the relative evidence for each model. According to the classification of Lee and Wagenmakers [37], a BF_10_>1 provides evidence in favor of the full model (ie, the alternative hypothesis), whereas a BF_10_<1 favors the null model.

#### Secondary Outcomes: Intervention Effects

We repeated the same Bayesian latent growth curve modeling approach to assess the effectiveness of the HRV and the EMAs we measured. Furthermore, we tested whether the FFMQ, neuroticism, and resilience had any impact on the self-reported stress reduction effect of the mindfulness condition versus the sham. To do so, we incorporated these individual difference measures as covariates in the model to examine their moderating effects on the trajectory of stress reduction over time. Specifically, we extended the latent growth curve model by adding interaction terms between the condition and each moderator (FFMQ, neuroticism, and resilience), and we estimated separate models for each moderator.

For each analysis, the full model (including the interaction term) was compared with a corresponding reduced model (excluding the interaction) using Bayes factors to quantify the evidence for a moderating effect. Notwithstanding the advantages of using Bayes factors in accommodating multiple tests [37], our analyses are potentially underpowered for detecting moderation effects when testing multiple outcomes; therefore, the results should be interpreted with caution.

Finally, the PARH scale was evaluated against its midpoint (4.0) using a 1-sample *t* test to assess demand characteristics [31].

## Results

### Demographics

**Figure 1:**
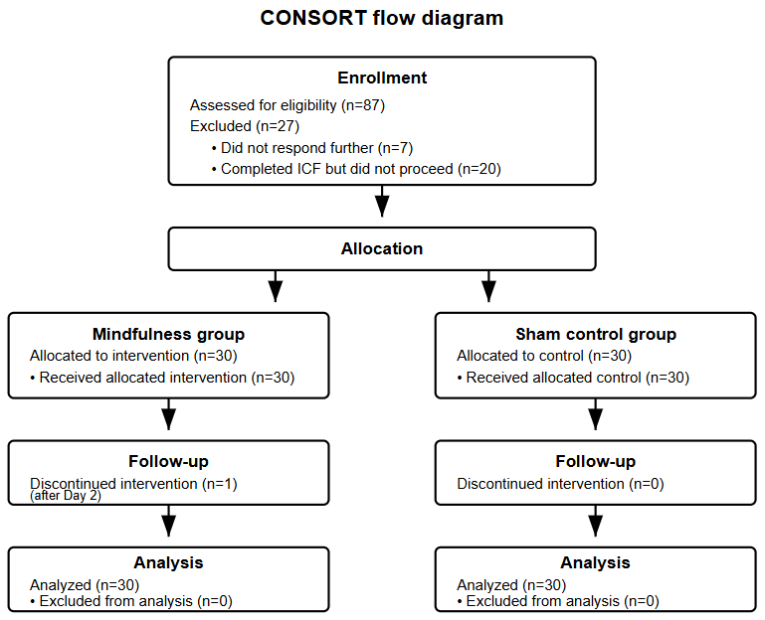
Consolidated Standards of Reporting Trials (CONSORT) diagram of study participants.

Participant recruitment began on February 12, 2025, and was completed on March 19, 2025. A total of 87 participants expressed interest in the study and completed the prescreening. Of these, 7 (8%) participants did not respond further, and 20 (23%) completed the informed consent form but did not proceed to the experiment. The remaining 60 (69%) participants were randomized into either the experimental or control group (refer to Figure 1 for the CONSORT flow diagram) [18]. All 60 (100%) participants were included in the final analysis. One (2%) participant discontinued the experiment after the end of day 2 but was retained in the analysis with available data. The remaining 59 (98%) participants completed the full study. The final sample comprised 60 participants (mean age 33.93, SD 12.18 years; range 21-70 years), with balanced sex representation (30/60, 50% female; 28/60, 47% male; and 2/60, 3% who preferred not to disclose). The ethnic composition reflected the regional demographics, with 93% (56/60) Chinese, 5% (3/60) Indian, and 2% (1/60) Malay participants. Random allocation successfully produced equal group sizes (n=30 per condition: mindfulness vs sham control) with no differences between groups in baseline characteristics (Table 1).

**Table 1:** Baseline demographic and psychological characteristics by experimental group (N=60)^a^.

Characteristic	Group			*P* value		Effect size, Cohen *d*
	Mindfulness (n=30)	Sham (n=30)				
Age (y), mean (SD)	36.20 (12)	31.60 (12.20)	.15		—^b^	
Female, n (%)	13 (43)	17 (57)	.39		—	
Chinese, n (%)	27 (90)	29 (97)	.61		—	
IPIP^c^, mean (SD)	2.75 (0.72)	2.94 (0.81)	.35		−0.24	
RS^d^, mean (SD)	5.16 (0.88)	5.06 (1.01)	.68		0.11	
FFMQ^e^, mean (SD)	2.94 (0.26)	2.86 (0.28)	.25		0.30	

^a^Effect sizes (Cohen *d*) are reported for continuous measures only. Key measures included the International Personality Item Pool (neuroticism subscale), the Resilience Scale, and the Five Facet Mindfulness Questionnaire. The mindfulness group (n=30) and the sham control group (n=30) showed no statistically significant differences in baseline characteristics (all *P*s>.05), suggesting successful randomization.

^b^Not applicable.

^c^IPIP: International Personality Item Pool.

^d^RS: Resilience Scale.

^e^FFMQ: Five Facet Mindfulness Questionnaire.

### Feasibility Outcomes

The decentralized trial design proved highly feasible, successfully recruiting 60 (69%) participants from the 87 participants screened, with near-perfect retention (59/60, 98%) completing all sessions. The participants demonstrated strong protocol adherence, with only 2% (1/60) of the participants discontinuing the experiment after the end of day 2. The HRV data collection was similarly robust, with 95% (57/60) of participants successfully submitting HRV files. Among the submitted measurements, 69.8% (231/331) met quality thresholds. The remaining 30.2% (100/331) of all collected PPG recordings were excluded from the analysis because of poor signal quality. The data quality assessment was performed by an internal algorithm that assesses the beat-to-beat variability of the derived RR intervals. Specifically, if the number of consecutive RR intervals deviating more than 25% from the preceding interval exceeded a predetermined threshold, the recording was flagged as low quality. A key factor in this data loss was the device’s operating system. Our analysis revealed a significant performance disparity between platforms. Of the total 187 recordings submitted from Android devices, 81 (43.3%) had poor signals. In contrast, of the 125 total recordings from iOS devices, 15 (12%) were reported to have a poor signal. For 19 recordings, device platform information was unavailable. These technical hurdles notwithstanding, the multimodal assessment protocol was successfully implemented in this remote context.

### Acceptability Findings

Quantitative usability ratings (1=*strongly disagree* to 5=*strongly agree*) indicated favorable evaluations overall (mean 4.17, SD 0.53), with consistently positive scores across the subscales: ease of access or navigation (mean 4.21, SD 0.66), clarity of instructions (mean 4.18, SD 0.69), comfort or engagement (mean 4.27, SD 0.57), and procedural acceptability (mean 4.14, SD 0.84). Notably, technical usability received slightly lower but still moderate ratings (mean 4.02, SD 0.64), aligning with qualitative reports of intermittent technical hurdles. Time commitment data revealed that most participants (42/60, 70%) spent approximately 30 to 40 minutes completing daily questionnaires, with similar daily time investment throughout the study. The most challenging aspects reported by participants included technical issues with the HRV measurement app (12/60, 20%), particularly overheating problems (eg, “flashlight burned my finger”); difficulty maintaining consistent timing and remembering to complete tasks (11/60, 18%) without reminders (eg, “sticking to the same time every day”); trouble focusing during meditation sessions (9/60, 15%); and navigating fragmented instructions (7/60, 12%; eg, “jumping back and forth between these 3 modes was quite distracting”). When asked about potential study improvements, 38% (23/60) of the participants offered no suggestions, while others recommended streamlining instructions (8/60, 13.3%) and implementing daily reminders (4/60, 6.7%). Regarding commitment willingness, most participants preferred short-term engagement: days to 1 week (27/60, 45%) or 2 weeks to 1 month (13/60, 21.7%), with only 3% (2/60) indicating openness to longer-term commitments. Despite the identified challenges, no adverse effects were reported, and the overall usability profile remained strong. For the full set of participant responses, refer to the project’s OSF repository [17].

### Stress and Physiological Outcomes

**Figure 2:**
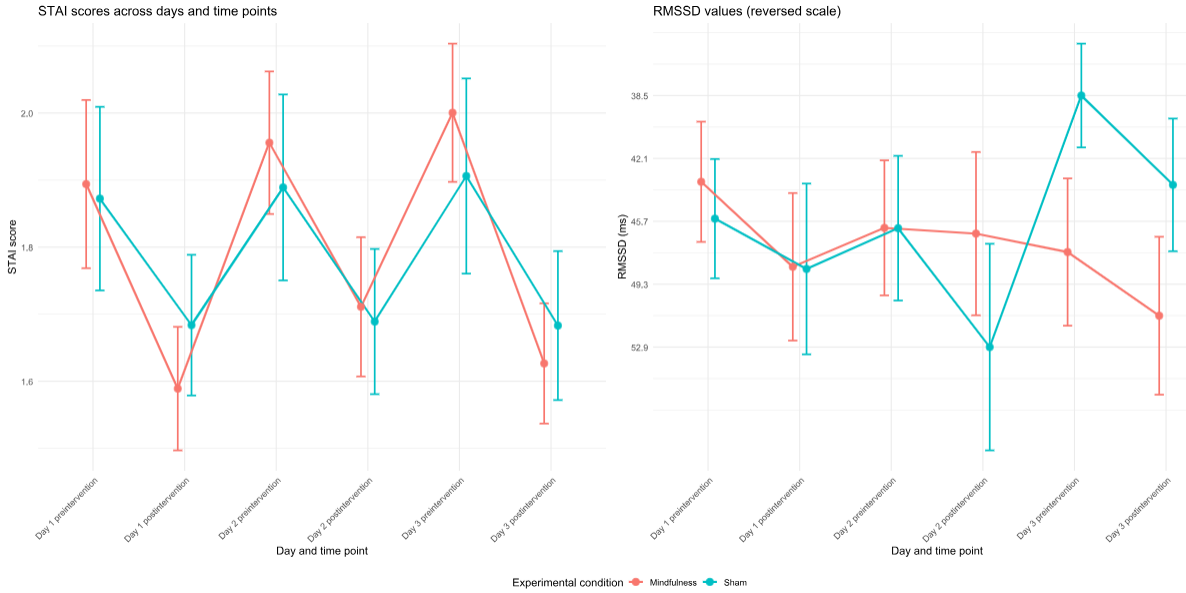
Changes in psychological and physiological stress measures across experimental days. Separate panels show (left) self-reported stress (State-Trait Anxiety Inventory; STAI) scores and (right) heart rate variability (root mean square of successive differences; RMSSD). For interpretive consistency, the RMSSD values are plotted on a reversed y-axis (ie, lower values appear higher on the plot) so that higher stress corresponds to higher plotted values in both panels. Data are presented in separate plots for clarity. The right panel displays RMSSD (heart rate variability in milliseconds) using a reversed y-axis. The left panel shows STAI scores with conventional orientation. In both panels, different colors represent experimental conditions, and error bars show plus or minus 1 standard error of the mean (SEM). Timepoints are arranged sequentially from left to right (day 1 preintervention, day 1 postintervention, day 2 preintervention, etc.), maintaining consistent x-axis scaling across plots to facilitate comparison.

Bayesian analyses revealed strong evidence for the null hypothesis for self-reported stress (STAI-6), indicating no differential improvement between conditions (BF_10_=0.03). For HRV (RMSSD), the results showed anecdotal evidence for the null hypothesis (BF_10_=0.20). Upon reviewer request, we conducted a prior sensitivity analysis to test the robustness of these findings. For the STAI-6 outcome, the Bayes factor remained strongly in favor of the null hypothesis with both a tighter, more skeptical prior (BF_10_=0.09) and a wider, more permissive prior (BF_10_<0.01). For RMSSD, the evidence remained anecdotal across the tighter (BF_10_=1.08) and wider (BF_10_=0.83) priors. However, both groups showed significant pre-to-post reductions in self-reported stress (BF_10_=3.01×10^6^). A moderate negative correlation emerged between RMSSD and self-reported stress (*r*=−0.28; *P*<.001), though their temporal patterns diverged during the intervention (Figure 2).

### EMA Findings

The same Bayesian latent growth curve models were applied to each EMA item (eg, “Right now, I feel fatigued” and “Right now, I feel stressed”). All analyses yielded inconclusive Bayes factors (BF_10_<1), indicating no statistically detectable differences between the mindfulness and sham meditation groups following experimental sessions for the EMAs scores.

### Demand Characteristic Effect

The analysis indicated that the mean PARH score (mean 4.10, SD 1.88) was not significantly different from the scale midpoint, t_56_=0.40, *P*=.689; 95% CI 3.60-4.60. This suggests that, on average, the participants did not report a clear awareness of the research hypotheses.

### Expectancy

**Figure 3:**
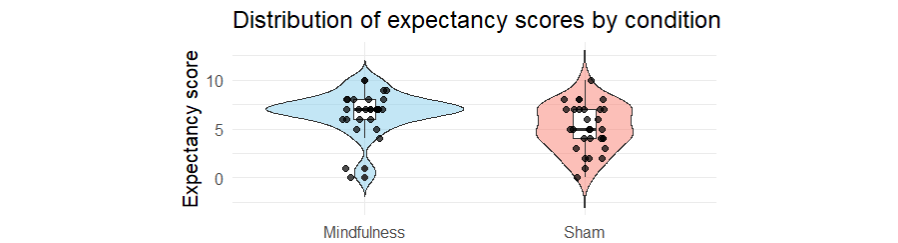
Distribution of expectancy scores by experimental condition.

The participants rated the perceived effectiveness of the received intervention on a scale from 0 (“not at all successful”) to 10 (“extremely successful”). The mindfulness condition yielded higher expectancy scores (mean 6.69, SD 2.19) compared with the sham condition (mean 5.13, SD 2.40), suggesting slightly stronger perceived effectiveness of the active intervention in alleviating stress. Figure 3 illustrates the full distribution of these scores, showing a higher median and a concentration of scores in the upper range for the mindfulness group. Given these differences, we conducted a covariate analysis to ensure that our primary findings were not an artifact of treatment expectancy. We adjusted our primary Bayesian mixed-effects model for the STAI by including baseline expectancy scores as a covariate. The results confirmed our original conclusion: after adjusting for expectancy, the analysis continued to show strong evidence for the null hypothesis of no differential improvement between the groups, yielding a Bayes factor of BF_10_=0.03.

### Credibility

The participants indicated which intervention they believed they had received (mindfulness or control) and rated their confidence (0-10). After excluding low-confidence responses (<5), credibility assessments revealed distinct patterns:

1. In the mindfulness group, 18 (75%) of 24 participants correctly identified their intervention, while 6 (25%) chose sham.
2. In the control (sham) group, 10 (48%) of 21 participants accurately selected sham, whereas 52% (11/21) incorrectly believed they had received mindfulness.

A chi-square test suggested a marginal but nonsignificant trend toward asymmetrical credibility perceptions between the groups, χ*^2^*_1_=3.2, *P*=.074. This aligns with the raw percentages, in which participants in the mindfulness group appeared more likely to correctly identify their condition, though the difference did not reach statistical significance.

### Exploratory Moderator Analyses

Examinations of baseline individual differences revealed no evidence for moderation effects on self-reported stress. The Bayes factor analyses indicated strong evidence in favor of the reduced models (ie, without moderation terms) over the full models that included interactions for trait mindfulness (FFMQ; BF_10_=0.009), neuroticism (BF_10_=0.116), and resilience (BF_10_=0.005). These findings suggest that the effects of the intervention on stress were not reliably moderated by these baseline traits. The null results may reflect limited statistical power to detect moderation effects in this pilot sample.

### Difference From Pre to Post

Bayesian latent growth curve modeling provided statistically significant evidence for reductions in self-reported stress, measured via the STAI-6 over time, regardless of the experimental condition. The full model, which included the time point predictor but did not account for the experimental condition, was compared with a null model containing only participant-level intercepts. This comparison yielded a Bayes factor (BF_10_) of 3.01×10^6^, strongly favoring the temporal model over the intercept-only null model. The results indicate that changes in the STAI-6 from before to after the assessment differed meaningfully across time points, with the full model strongly supported over the null. Importantly, this analysis did not consider the experimental condition, suggesting that the observed before-and-after differences in self-reported stress levels occurred irrespective of the specific experimental manipulation. Descriptive statistics for STAI-6 scores across days and time points for both conditions are presented in Table 2.

**Table 2:** Mean State-Trait Anxiety Inventory scores by condition and time point.

Time and condition		Day 1, mean (SD)	Day 2, mean (SD)	Day 3, mean (SD)
**Pre**				
	Mindfulness (n=30)	1.89 (0.69)	1.96 (0.58)	2 (0.57)
	Sham (n=30)	1.87 (0.75)	1.89 (0.76)	1.91 (0.80)
**Post**				
	Mindfulness (n=30)	1.59 (0.50)	1.71 (0.57)	1.63 (0.49)
	Sham (n=30)	1.68 (0.58)	1.69 (0.59)	1.68 (0.61)

## Discussion

### Principal Findings

This pilot trial addressed several key methodological gaps in digital mindfulness research by evaluating the feasibility and acceptability of a fully decentralized SAM intervention. Building on prior work, this study incorporated 3 methodological innovations: a structurally matched sham control, smartphone-based HRV as an objective biomarker, and fully remote delivery via Qualtrics and mobile platforms.

To our knowledge, this is the first mindfulness trial to measure the stress response using both self-reported and physiological measures in a fully decentralized setting, while using a rigorous control condition to disentangle true intervention effects from any demand characteristic or expectancy effects of participants. The design of the brief, 3-day intervention was a deliberate balance between established mindfulness principles and the practical constraints of a decentralized pilot study. It was directly informed by a formative focus group with expert mindfulness instructors, who recommended focusing on foundational and accessible practices—such as breath and body awareness—as the most suitable content for beginners in a remote setting. The 10-minute session length was chosen based on expert consensus that this duration is sufficient to be impactful yet brief enough to maximize daily adherence. While a 3-day intervention is not intended to produce lasting clinical change, it was deemed an appropriate timeframe to robustly test the feasibility of the novel, fully remote protocol and to observe initial stress responses.

The findings strongly support the feasibility and acceptability of the protocol, with high retention rates, strong adherence to daily procedures, successful collection of HRV and self-reported assessments, and high usability ratings. This study lays a strong methodological foundation for scaling up decentralized mindfulness trials. Nonetheless, although our decentralized approach successfully enabled remote data collection of self-report and physiological measures, our findings raise some concerns about the efficacy of the intervention and the measurement accuracy of the smartphone-based HRV app.

### Feasibility of Decentralized Delivery

This study demonstrated strong feasibility for a decentralized delivery model, with a participant retention rate of 98.3%, which surpasses established benchmarks. Protocol adherence was high overall; the participants independently completed all study procedures, including HRV recordings, online surveys, and audio-guided sessions with minimal supervision. A key feasibility outcome was the self-administration of smartphone-based PPG for HRV.

While 95% (57/60) of participants successfully submitted HRV data, only 69.8% (42/60) of these recordings met predefined quality thresholds, resulting in a 30.2% data exclusion rate. These signal artifacts were likely attributable to a combination of improper finger placement and significant device-specific performance differences. To contextualize this, the published literature on the Camera HRV app reports data availability rates from 75% to more than 90% in controlled conditions, which can drop significantly in real-world settings [27]. Therefore, while our valid data yield is on the lower end, it is not outside the range reported for ecologically valid recordings. Nevertheless, these results support PPG as a viable, though imperfect, tool for remote monitoring [38]. The observed challenges underscore the need for enhanced onboarding materials, such as step-by-step instructional videos or in-app quality checks, especially to mitigate data loss on the Android platform [39].

### Acceptability and Participant Experience

This study demonstrated strong evidence of acceptability across multiple evaluation approaches. The participants generally found the study materials, delivery platforms, and procedures accessible and manageable, as reflected in both quantitative ratings and qualitative feedback. While the usability scores consistently met or exceeded our predefined benchmarks, the more nuanced qualitative data revealed important contextual factors that shaped the participants’ experiences.

A key strength emerged in participants’ overall positive perception of the protocol’s structure and requirements. The high ratings across various usability dimensions—including ease of navigation, clarity of instruction, and procedural comfort—suggest that the core design elements were well conceived and appropriately implemented for remote delivery. This was particularly noteworthy given the technical demands of self-administered physiological measurements in a decentralized setting.

However, the participant feedback illuminated several challenges that warrant attention. Technical difficulties with the HRV measurement emerged as a prominent concern, with multiple reports of inconsistent readings and physical discomfort owing to device overheating.

The qualitative data also highlighted that certain protocol requirements—while theoretically straightforward—proved challenging in practice. Maintaining consistent measurement times without automated reminders emerged as an unexpected burden for some participants. Similarly, the need to navigate between multiple platforms and sets of instructions created friction points that, while not fundamentally undermining participation, added to the cognitive load of the protocol.

These findings carry important implications for future implementations. The technical issues suggest a need for additional participant guidance to ensure consistent data collection. The feedback regarding scheduling and platform navigation highlights opportunities to streamline procedures and integrate more participant support mechanisms. Notably, these refinements would not require fundamental changes to the study design, but rather targeted improvements to the user experience. The convergence of generally positive usability ratings with specific, actionable feedback creates a valuable road map for optimization. The participants’ ability to articulate both what worked well and where they encountered difficulties speaks to the overall thoughtfulness of the protocol while highlighting areas where the user experience could be enhanced. This balance between overall acceptability and identifiable improvement opportunities is particularly encouraging for the potential scalability of the approach.

### Mindfulness Intervention and Sham Condition: Parsing Specific Versus Nonspecific Effects

As a feasibility pilot, the trial was not powered to detect small effects in psychological or physiological outcomes. Nevertheless, both the mindfulness and the sham groups exhibited significant pre-post reductions in self-reported stress, consistent with the notion that engaging in structured, reflective practices, regardless of specific content, can yield immediate benefits. These findings align with prior studies showing that even brief interventions can produce short-term stress relief [6].

The absence of between-group differences may reflect the brevity of the intervention, 3 audio-guided sessions delivered over 3 consecutive days, which likely provided insufficient exposure for participants to cultivate foundational mindfulness skills, such as sustained attentional focus and metacognitive awareness. Previous research suggests that mindfulness-related neuroplastic and physiological changes typically emerge after several weeks of consistent practice [40]. Thus, while the observed reductions in stress support the efficacy of brief contemplative practices, they also highlight the limitations of short-term interventions for isolating mindfulness-specific mechanisms from nonspecific or expectancy-driven effects.

Concerns about demand characteristics were partially addressed through PARH scores [32], which did not significantly deviate from the scale midpoint. This suggests that participants had limited explicit awareness of the study’s hypotheses, mitigating concerns about biased responding. Notably, the modest correlation between HRV and self-reported stress underscores partial convergence between subjective and objective stress measures [41], while also revealing their distinct sensitivity to brief interventions. These results reinforce the value of combining multimodal assessments in stress research, while also illustrating the interpretive challenges associated with physiological metrics in short-term, real-world contexts. Whether longer or more intensive interventions would produce stronger alignment between physiological and self-reported outcomes remains an open and important question.

### Implications for Sham Control Design

A central methodological contribution of this study was the development and deployment of a structurally matched sham meditation condition, aimed at isolating mindfulness-specific effects by controlling for nonspecific influences, such as expectancy and demand characteristics. Both groups reported comparable levels of expectancy and perceived credibility; however, participants in the mindfulness condition were more accurate in identifying their assigned intervention and expressed greater confidence in their judgments. Although the differences in credibility ratings were not statistically significant, this asymmetry suggests that the sham condition may not have achieved full perceptual equivalence regarding legitimacy or engagement. This reflects a well-documented challenge in mindfulness research: creating control conditions that are structurally and psychologically comparable without inadvertently activating therapeutic mechanisms [42].

While our active sham was designed to omit the core components of mindfulness (eg, meta-awareness and attentional anchoring), it still contained nonspecific therapeutic factors common to both conditions, such as taking a dedicated break, listening to a calming voice, and having an expectation of benefit. For a broad outcome, such as stress, these nonspecific elements may play a role. This “tricky task” of sham design is further complicated by individual differences; some participants may find the “mind-wandering” exercises of a sham condition inherently relaxing, while others may not. This underscores that, although our design successfully controlled for the specific process of mindfulness, future research must continue to develop more sophisticated controls to fully disentangle the specific versus nonspecific drivers of stress reduction in remote interventions.

Furthermore, although effective in excluding core mindfulness components, the design may have inadvertently reduced its plausibility as a credible intervention. Establishing a truly credible and inert sham control is not merely a methodological ideal; it is essential for drawing valid inferences about mindfulness-specific mechanisms. Without adequate control for expectancy and engagement, intervention effects risk being confounded by demand characteristics, thereby complicating interpretation and potentially inflating perceived efficacy. This pilot study identified the need for further refinement and validation of the sham condition before launching a full-scale intervention.

### Decentralized Design: Trade-Offs Between Scalability and Precision

While this study underscores the promise of remote, smartphone-based interventions, it also highlights key technical and logistical challenges that warrant iterative refinement. The relatively high rate of poor-quality physiological data points to issues, such as device compatibility, signal variability, and user interface limitations—challenges that could be mitigated through the open sharing of troubleshooting protocols and device testing datasets (refer to Table 3, “Open science”). Addressing these hurdles will require improved app functionality (eg, features to prevent overheating during PPG recordings) alongside transparent reporting of technical failures to inform future development. Participant feedback emphasized difficulties with fixed schedules (“sticking to the same time every day”) and fragmented platforms (“jumping back and forth between these 3 modes was quite distracting”). To accelerate solutions for such barriers, we advocate for open repositories of usability feedback and deidentified adherence data resources to help standardize best practices across studies.

**Table 3:** Key recommendations for optimizing fully decentralized digital health studies^a^.

Domain	Recommendation	Key benefit
Preregistration	Preregister protocols (eg, ClinicalTrials.gov and OSF^b^)	Ensures transparency, reduces bias, and clarifies hypotheses upfront
Open science	Share deidentified data (where ethically appropriate), code, and materials openly	Enables replication and accelerates research progress
Training	Use video tutorials for technical tasks	Minimizes technical errors, enhances adherence, and promotes participant autonomy by reducing the need for coordinator support
Scheduling	Allow flexible timing for participant tasks	Boosts adherence by accommodating real-world schedules
Automation	Send automated reminders (SMS text messaging or email)	Lowers missed assessments without staff effort
Platform design	Integrate all tools into 1 user-friendly system	Prevents confusion and streamlines user experience
Measurements	Focus only on essential outcomes	Reduces participant burden and dropout rates, and maintains statistical power
Testing	Test devices rigorously before launch	Avoids technical failures (eg, unstable signals and data loss)
Control conditions	Use credible, matched active control groups	Isolates true treatment effects from expectation or demand characteristic effects

^a^This checklist outlines practical design considerations for researchers conducting decentralized clinical trials, with specific emphasis on enhancing feasibility, participant adherence, and data quality by addressing common logistical and technical challenges.

^b^OSF: Open Science Framework.

Despite these hurdles, the high usability ratings and strong retention demonstrate the feasibility and appeal of decentralized mindfulness interventions when designed with the end-user experience in mind. The scalability of decentralized methods remains their defining strength, enabling access to diverse populations in ecologically valid settings. To support broader adoption, we provide a practical checklist (Table 3) that synthesizes lessons from this trial, with open science practices embedded as a core pillar for enhancing reproducibility and collaborative problem-solving.

### Limitations and Constraints on Generality

Several limitations temper the interpretation of our findings. First, while the sample size (n=60) was adequate for assessing feasibility and acceptability [20], it was underpowered to detect subtle between-group differences or moderation effects. Second, the total guided practice time was notably brief, amounting to only 30 minutes across 3 sessions. This minimal dosage, while practical for a feasibility trial, may have been sufficient only to capture immediate, short-term effects, rather than to induce substantial or lasting changes in well-being. In addition, the lack of follow-up assessments precludes evaluation of the intervention’s long-term effects. Third, this study had constrained generalizability [43]. The findings are most applicable to (1) English-fluent adults (aged >21 years) in Singapore, (2) nonmeditators (≥6 months) without mental health histories or medication use, and (3) predominantly Chinese Singaporean participants (56/60, 93.3%). Cultural differences in stress expression and coping may limit applicability to broader populations. Fourth, in line with the aim of the study to test a flexible and ecologically valid decentralized intervention, we did not mandate a specific time of day for the daily HRV assessments. Although participants were encouraged to complete these sessions at a consistent time to minimize circadian effects, we could not enforce or verify their adherence. Therefore, unmeasured circadian variations in HRV are a limitation of this study and may have introduced additional noise into the physiological data.

### Conclusions

This pilot study advances decentralized mental health research by demonstrating the feasibility of a fully remote mindfulness intervention incorporating smartphone-based physiological monitoring. The protocol achieved exceptional retention and acceptable, fully remote HRV data quality, supporting the viability of decentralized designs for multimodal stress assessment. Although both the mindfulness and sham groups showed comparable reductions in stress, highlighting the challenge of isolating specific effects in brief interventions, the modest correlation between HRV and self-reported measures underscores the value of combined physiological and psychological assessment. Three critical lessons emerge: (1) technical refinements (eg, onboarding videos and device testing) could improve the reliability of remote HRV data collection; (2) longer intervention durations may be necessary to distinguish mindfulness-specific mechanisms from demand characteristic or expectation effects; and (3) the sham meditation condition requires further optimization to balance credibility with mechanistic specificity. These findings provide a methodological road map for scaling decentralized trials while highlighting the need for open science approaches to address technical and design challenges. Future research should extend these findings to larger, more diverse samples with longer-term follow-up, leveraging decentralized designs to bridge the gap between rigorously controlled research and real-world implementation.

## Appendix

Multimedia Appendix 1 CONSORT-eHEALTH checklist (V 1.6.1).

## Abbreviations

BF10: Bayes factor10
ECG: electrocardiogram
CONSORT: Consolidated Standards of Reporting Trials
CONSORT-EHEALTH: Consolidated Standards of Reporting Trials of Electronic and Mobile Health Applications and Online Telehealth
EMA: ecologic momentary assessment
FFMQ: Five Facet Mindfulness Questionnaire
HRV: heart rate variability
MBSR: mindfulness-based stress reduction
OSF: Open Science Framework
PARH: Perceived Awareness Of The Research Hypothesis
PPG: photoplethysmography
RMSSD: root mean square of successive differences
SAM: self-administered mindfulness
STAI: State-Trait Anxiety Inventory
SUS: System Usability Scale

## Data Availability

The data and analysis scripts for the 42 participants who provided consent for data sharing are publicly available in the OSF repository [17] under a Creative Commons Attribution 4.0 International License. The data for the remaining 18 participants are not available due to a lack of consent for public sharing. Please note that because the shared data represent a subset of the full sample, re-running the analysis scripts may yield slightly different results from those reported in the manuscript.
