# Supplementary material for: Feasibility and Acceptability of a Smartphone-Delivered Mindfulness Intervention for Stress Reduction in Adult Singaporeans: Pilot Randomized Controlled Trial
Source: JMIR Ment Health. 2025 Aug 19;12:e77793. doi: 10.2196/77793 (PMC12405792; doi:10.2196/77793)
Supplement: Multimedia Appendix 1 [file mental_v12i1e77793_app1.pdf]

CONSORT-EHEALTH (V 1.6.1) - Submission/Publication Form

The CONSORT-EHEALTH checklist is intended for authors of randomized trials evaluating web-based and Internet-based applications/interventions, including mobile interventions, electronic games (incl multiplayer games), social media, certain telehealth applications, and other interactive and/or networked electronic applications. Some of the items (e.g. all subitems under item 5 - description of the intervention) may also be applicable for other study designs.

The goal of the CONSORT EHEALTH checklist and guideline is to be
a) a guide for reporting for authors of RCTs,
b) to form a basis for appraisal of an ehealth trial (in terms of validity)

CONSORT-EHEALTH items/subitems are MANDATORY reporting items for studies published in the Journal of Medical Internet Research and other journals / scientific societies endorsing the checklist.

Items numbered 1., 2., 3., 4a., 4b etc are original CONSORT or CONSORT-NPT (non-pharmacologic treatment) items.
Items with Roman numerals (i., ii, iii, iv etc.) are CONSORT-EHEALTH extensions/clarifications.

As the CONSORT-EHEALTH checklist is still considered in a formative stage, we would ask that you also RATE ON A SCALE OF 1-5 how important/useful you feel each item is FOR THE PURPOSE OF THE CHECKLIST and reporting guideline (optional).

Mandatory reporting items are marked with a red *.
In the textboxes, either copy & paste the relevant sections from your manuscript into this form - please include any quotes from your manuscript in QUOTATION MARKS,
or answer directly by providing additional information not in the manuscript, or elaborating on why the item was not relevant for this study.

YOUR ANSWERS WILL BE PUBLISHED AS A SUPPLEMENTARY FILE TO YOUR PUBLICATION IN JMIR AND ARE CONSIDERED PART OF YOUR PUBLICATION (IF ACCEPTED).
Please fill in these questions diligently. Information will not be copyedited, so please use proper spelling and grammar, use correct capitalization, and avoid abbreviations.

DO NOT FORGET TO SAVE AS PDF _AND_ CLICK THE SUBMIT BUTTON SO YOUR ANSWERS ARE IN OUR DATABASE !!!

Citation Suggestion (if you append the pdf as Appendix we suggest to cite this paper in the caption):
Eysenbach G, CONSORT-EHEALTH Group
CONSORT-EHEALTH: Improving and Standardizing Evaluation Reports of Web-based and Mobile Health Interventions
J Med Internet Res 2011;13(4):e126

URL: http://www.jmir.org/2011/4/e126/
doi: 10.2196/jmir.1923
PMID: 22209829

ale.sparacio92@gmail.com Cambia account

Bozza non salvata

Non condiviso

* Indica una domanda obbligatoria

Your name *

First Last

Alessandro Sparacio

Primary Affiliation (short), City, Country *

University of Toronto, Toronto, Canada

Institute for Human Development and Potentia

Your e-mail address *

abc@gmail.com

ale.sparacio92@gmail.com

Title of your manuscript *

Provide the (draft) title of your manuscript.

Feasibility and Acceptability of a Smartphone-Delivered Mindfulness Intervention for Stress Reduction in Adult Singaporeans: A Pilot Randomized Controlled Trial.

Name of your App/Software/Intervention *

If there is a short and a long/alternate name, write the short name first and add the long name in brackets.

Self-administered Mindfulness

Evaluated Version (if any)

e.g. "V1", "Release 2017-03-01", "Version 2.0.27913"

Version 1.0, Release 2025-16-5

Language(s) *

What language is the intervention/app in? If multiple languages are available, separate by comma (e.g. "English, French")

English

URL of your Intervention Website or App

e.g. a direct link to the mobile app on app in appstore (itunes, Google Play), or URL of the website. If the intervention is a DVD or hardware, you can also link to an Amazon page.

https://apps.apple.com/us/app/camera-heart-rate-variability/id788460316

URL of an image/screenshot (optional)

La tua risposta

Accessibility *

Can an enduser access the intervention presently?

- (selected) access is free and open
- access only for special usergroups, not open
- access is open to everyone, but requires payment/subscription/in-app purchases
- app/intervention no longer accessible
- Altro:

Primary Medical Indication/Disease/Condition *

e.g. "Stress", "Diabetes", or define the target group in brackets after the condition, e.g. "Autism (Parents of children with)", "Alzheimers (Informal Caregivers of)"

Stress

Primary Outcomes measured in trial *

comma-separated list of primary outcomes reported in the trial

Feasibility, Acceptability, Self-reported stress ([illegible]

Secondary/other outcomes

Are there any other outcomes the intervention is expected to affect?

'Secondary objectives explored (1) HRV as a physiological stress marker, (2) Ecological Momentary Assessments (EMA)-derived transient states (e.g., fatigue), and (3) moderation by baseline traits (FFMQ, resilience, neuroticism).'

Recommended "Dose" *

What do the instructions for users say on how often the app should be used?

- (•) Approximately Daily
- ( ) Approximately Weekly
- ( ) Approximately Monthly
- ( ) Approximately Yearly
- ( ) "as needed"
- ( ) Altro:

Approx. Percentage of Users (starters) still using the app as recommended after 3 months *

- (•) unknown / not evaluated
- ( ) 0-10%
- ( ) 11-20%
- ( ) 21-30%
- ( ) 31-40%
- ( ) 41-50%
- ( ) 51-60%
- ( ) 61-70%
- ( ) 71%-80%
- ( ) 81-90%
- ( ) 91-100%
- ( ) Altro:

Overall, was the app/intervention effective? *

- ( ) yes: all primary outcomes were significantly better in intervention group vs control
- ( ) partly: SOME primary outcomes were significantly better in intervention group vs control
- (x) no statistically significant difference between control and intervention
- ( ) potentially harmful: control was significantly better than intervention in one or more outcomes
- ( ) inconclusive: more research is needed
- ( ) Altro:

Article Preparation Status/Stage *

At which stage in your article preparation are you currently (at the time you fill in this form)

- ( ) not submitted yet - in early draft status
- ( ) not submitted yet - in late draft status, just before submission
- ( ) submitted to a journal but not reviewed yet
- (x) submitted to a journal and after receiving initial reviewer comments
- ( ) submitted to a journal and accepted, but not published yet
- ( ) published
- ( ) Altro:

Journal *

If you already know where you will submit this paper (or if it is already submitted), please provide the journal name (if it is not JMIR, provide the journal name under "other")

- ○ not submitted yet / unclear where I will submit this
- ○ Journal of Medical Internet Research (JMIR)
- ○ JMIR mHealth and UHealth
- ○ JMIR Serious Games
- ○ JMIR Mental Health
- ○ JMIR Public Health
- ○ JMIR Formative Research
- ○ Other JMIR sister journal
- ◉ Altro: Pending editor approval for JMIR Mental Health

Is this a full powered effectiveness trial or a pilot/feasibility trial? *

- ◉ Pilot/feasibility
- ○ Fully powered

Manuscript tracking number *

If this is a JMIR submission, please provide the manuscript tracking number under "other" (The ms tracking number can be found in the submission acknowledgement email, or when you login as author in JMIR. If the paper is already published in JMIR, then the ms tracking number is the four-digit number at the end of the DOI, to be found at the bottom of each published article in JMIR)

- ○ no ms number (yet) / not (yet) submitted to / published in JMIR
- ◉ Altro: #77793

TITLE AND ABSTRACT

1a) TITLE: Identification as a randomized trial in the title

1a) Does your paper address CONSORT item 1a? *

I.e does the title contain the phrase "Randomized Controlled Trial"? (if not, explain the reason under "other")

- (x) yes
- ( ) Altro:

1a-i) Identify the mode of delivery in the title

Identify the mode of delivery. Preferably use “web-based” and/or “mobile” and/or “electronic game” in the title. Avoid ambiguous terms like “online”, “virtual”, “interactive”. Use “Internet-based” only if Intervention includes non-web-based Internet components (e.g. email), use “computer-based” or “electronic” only if offline products are used. Use “virtual” only in the context of “virtual reality” (3-D worlds). Use “online” only in the context of “online support groups”. Complement or substitute product names with broader terms for the class of products (such as “mobile” or “smart phone” instead of “iphone”), especially if the application runs on different platforms.

	1	2	3	4	5	
subitem not at all important	( )	( )	( )	(x)	( )	essential

Cancella selezione

Does your paper address subitem 1a-i? *

Copy and paste relevant sections from manuscript title (include quotes in quotation marks "like this" to indicate direct quotes from your manuscript), or elaborate on this item by providing additional information not in the ms, or briefly explain why the item is not applicable/relevant for your study

'Feasibility and Acceptability of a Smartphone-Delivered Mindfulness Intervention for Stress Reduction in Adult Singaporeans: A Pilot Randomized Controlled Trial'

1a-ii) Non-web-based components or important co-interventions in title

Mention non-web-based components or important co-interventions in title, if any (e.g., “with telephone support”).

	1	2	3	4	5	
subitem not at all important	○	○	◉	○	○	essential

Cancella selezione

Does your paper address subitem 1a-ii?

Copy and paste relevant sections from manuscript title (include quotes in quotation marks "like this" to indicate direct quotes from your manuscript), or elaborate on this item by providing additional information not in the ms, or briefly explain why the item is not applicable/relevant for your study

not relevant for this study.

1a-iii) Primary condition or target group in the title

Mention primary condition or target group in the title, if any (e.g., “for children with Type I Diabetes”) Example: A Web-based and Mobile Intervention with Telephone Support for Children with Type I Diabetes: Randomized Controlled Trial

	1	2	3	4	5	
subitem not at all important	○	○	○	◉	○	essential

Cancella selezione

Does your paper address subitem 1a-iii? *

Copy and paste relevant sections from manuscript title (include quotes in quotation marks "like this" to indicate direct quotes from your manuscript), or elaborate on this item by providing additional information not in the ms, or briefly explain why the item is not applicable/relevant for your study

we refer to the target population 'Adult Singaporeans' in the title.

1b) ABSTRACT: Structured summary of trial design, methods, results, and conclusions

NPT extension: Description of experimental treatment, comparator, care providers, centers, and blinding status.

1b-i) Key features/functionalities/components of the intervention and comparator in the METHODS section of the ABSTRACT

Mention key features/functionalities/components of the intervention and comparator in the abstract. If possible, also mention theories and principles used for designing the site. Keep in mind the needs of systematic reviewers and indexers by including important synonyms. (Note: Only report in the abstract what the main paper is reporting. If this information is missing from the main body of text, consider adding it)

	1	2	3	4	5	
subitem not at all important	○	○	○	○	◉	essential

Cancella selezione

Does your paper address subitem 1b-i? *

Copy and paste relevant sections from the manuscript abstract (include quotes in quotation marks "like this" to indicate direct quotes from your manuscript), or elaborate on this item by providing additional information not in the ms, or briefly explain why the item is not applicable/relevant for your study

We provided all the information requested in the method section of the Abstract: ' Methods: This was a purely smartphone-based, decentralized pilot trial with no face-to-face components. Participants were recruited online and, after providing informed consent, sixty adults were randomized to either a mindfulness intervention or a structurally matched sham control. The daily 10-minute mindfulness sessions guided participants to focus on present-moment breath and body sensations, while the sham control omitted a focus on present-moment awareness. Outcomes were assessed remotely, using self-report questionnaires (STAI-6) and physiological data from smartphone-based photoplethysmography (Heart Rate Variability; HRV). The study incorporated three methodological innovations: (1) a structurally equivalent sham control to match expectancy and credibility, (2) remote collection of HRV as an objective physiological biomarker, and (3) full decentralization allowing unsupervised multi-platform delivery. Feasibility was evaluated through recruitment/retention rates and data quality. Acceptability was assessed through quantitative ratings and qualitative feedback.

1b-ii) Level of human involvement in the METHODS section of the ABSTRACT

Clarify the level of human involvement in the abstract, e.g., use phrases like “fully automated” vs. “therapist/nurse/care provider/physician-assisted” (mention number and expertise of providers involved, if any). (Note: Only report in the abstract what the main paper is reporting. If this information is missing from the main body of text, consider adding it)

	1	2	3	4	5	
subitem not at all important	◉	○	○	○	○	essential

Cancella selezione

Does your paper address subitem 1b-ii?

Copy and paste relevant sections from the manuscript abstract (include quotes in quotation marks "like this" to indicate direct quotes from your manuscript), or elaborate on this item by providing additional information not in the ms, or briefly explain why the item is not applicable/relevant for your study

We specified that the study required minimal supervision: 'This pre-registered pilot study (NCT06765889) evaluated a decentralized, three-day self-administered mindfulness intervention with minimal supervision'

1b-iii) Open vs. closed, web-based (self-assessment) vs. face-to-face assessments in the METHODS section of the ABSTRACT

Mention how participants were recruited (online vs. offline), e.g., from an open access website or from a clinic or a closed online user group (closed usergroup trial), and clarify if this was a purely web-based trial, or there were face-to-face components (as part of the intervention or for assessment). Clearly say if outcomes were self-assessed through questionnaires (as common in web-based trials). Note: In traditional offline trials, an open trial (open-label trial) is a type of clinical trial in which both the researchers and participants know which treatment is being administered. To avoid confusion, use "blinded" or "unblinded" to indicated the level of blinding instead of "open", as "open" in web-based trials usually refers to "open access" (i.e. participants can self-enrol). (Note: Only report in the abstract what the main paper is reporting. If this information is missing from the main body of text, consider adding it)

	1	2	3	4	5	
subitem not at all important	○	◉	○	○	○	essential

Cancella selezione

Does your paper address subitem 1b-iii?

Copy and paste relevant sections from the manuscript abstract (include quotes in quotation marks "like this" to indicate direct quotes from your manuscript), or elaborate on this item by providing additional information not in the ms, or briefly explain why the item is not applicable/relevant for your study

'This was a purely smartphone-based, decentralized pilot trial with no face-to-face components. Participants were recruited online and, after providing informed consent, sixty adults were randomized to either a mindfulness intervention or a structurally matched sham control. The daily 10-minute mindfulness sessions guided participants to focus on present-moment breath and body sensations, while the sham control omitted a focus on present-moment awareness. Outcomes were assessed remotely, using self-report questionnaires (STAI-6) and physiological data from smartphone-based photoplethysmography (Heart Rate Variability; HRV). The study incorporated three methodological innovations: (1) a structurally equivalent sham control to match expectancy and credibility, (2) remote collection of HRV as an objective physiological biomarker, and (3) full decentralization allowing unsupervised multi-platform delivery. Feasibility was evaluated through recruitment/retention rates and data quality. Acceptability was assessed through quantitative ratings and qualitative feedback.'

1b-iv) RESULTS section in abstract must contain use data

Report number of participants enrolled/assessed in each group, the use/uptake of the intervention (e.g., attrition/adherence metrics, use over time, number of logins etc.), in addition to primary/secondary outcomes. (Note: Only report in the abstract what the main paper is reporting. If this information is missing from the main body of text, consider adding it)

	1	2	3	4	5	
subitem not at all important	○	○	◉	○	○	essential

Cancella selezione

Does your paper address subitem 1b-iv?

Copy and paste relevant sections from the manuscript abstract (include quotes in quotation marks "like this" to indicate direct quotes from your manuscript), or elaborate on this item by providing additional information not in the ms, or briefly explain why the item is not applicable/relevant for your study

'The study demonstrated excellent feasibility with near perfect retention (98.3%) and moderate HRV data quality (n = 231, 69.8% valid signals). Acceptability ratings were strong (M = 4.17/5, SD = 0.53), with highest scores for comfort/engagement (M = 4.27/5, SD = 0.57), exceeding established usability benchmarks for digital health interventions. Qualitative feedback identified technical challenges (HRV instability, device overheating) and scheduling difficulties. While Bayesian analyses did not detect significant group differences in stress reduction (BF_{10} = 0.03) or HRV improvement (BF_{10} = 0.2), both groups showed significant stress reductions (BF_{10}= 3.01×10^6), suggesting that observed benefits may stem from non-specific factors common to both interventions.'

1b-v) CONCLUSIONS/DISCUSSION in abstract for negative trials

Conclusions/Discussions in abstract for negative trials: Discuss the primary outcome - if the trial is negative (primary outcome not changed), and the intervention was not used, discuss whether negative results are attributable to lack of uptake and discuss reasons. (Note: Only report in the abstract what the main paper is reporting. If this information is missing from the main body of text, consider adding it)

	1	2	3	4	5	
subitem not at all important	○	○	○	●	○	essential

Cancella selezione

Does your paper address subitem 1b-v?

Copy and paste relevant sections from the manuscript abstract (include quotes in quotation marks "like this" to indicate direct quotes from your manuscript), or elaborate on this item by providing additional information not in the ms, or briefly explain why the item is not applicable/relevant for your study

'Conclusions: This study demonstrates (1) the feasibility of conducting fully decentralized mindfulness trials with multimodal assessment, (2) the value of mixed-methods acceptability evaluation, and (3) identifies key technical and control condition refinements necessary for future trials. By addressing methodological limitations through improved control conditions and objective measures, this work provides a foundation for more rigorous investigation of mindfulness-specific effects. The findings suggest that both the brief mindfulness and sham interventions require adaptation, alongside technical improvements, to be effective in future, potentially longer, trials.'

INTRODUCTION

2a) In INTRODUCTION: Scientific background and explanation of rationale

2a-i) Problem and the type of system/solution

Describe the problem and the type of system/solution that is object of the study: intended as stand-alone intervention vs. incorporated in broader health care program? Intended for a particular patient population? Goals of the intervention, e.g., being more cost-effective to other interventions, replace or complement other solutions? (Note: Details about the intervention are provided in “Methods” under 5)

	1	2	3	4	5	
subitem not at all important	○	○	◉	○	○	essential

Cancella selezione

Does your paper address subitem 2a-i? *

Copy and paste relevant sections from the manuscript (include quotes in quotation marks "like this" to indicate direct quotes from your manuscript), or elaborate on this item by providing additional information not in the ms, or briefly explain why the item is not applicable/relevant for your study

'Stress exerts a pervasive influence on psychological and physiological wellbeing, contributing to conditions such as depression and anxiety [1,2,3]. In Singapore, mental health concerns rank as the top healthcare priority among residents (46%), ahead of cancer (38%) and stress-related issues (35%) [4]. The economic costs are also considerable: Symptoms of depression and anxiety are estimated to account for a 2.9% loss of Singapore’s GDP. Addressing this burden requires interventions that are not only effective but also scalable, accessible, and acceptable to diverse populations outside of traditional clinical settings.

Mindfulness - particularly in digital, self-administered formats - has emerged as a promising strategy for supporting mental health at scale, with a growing body of evidence linking it to reductions in stress, anxiety, and emotional dysregulation [5,6]. In particular, self-administered mindfulness (SAM) interventions - which remove the need for trained instructors and in-person delivery - offer practical advantages in terms of reach, cost-effectiveness, and ecological validity [7]. However, concerns remain about the strength of evidence for such interventions, particularly when delivered digitally and evaluated in uncontrolled or self-selected samples.'

2a-ii) Scientific background, rationale: What is known about the (type of) system

Scientific background, rationale: What is known about the (type of) system that is the object of the study (be sure to discuss the use of similar systems for other conditions/diagnoses, if appropiate), motivation for the study, i.e. what are the reasons for and what is the context for this specific study, from which stakeholder viewpoint is the study performed, potential impact of findings [2]. Briefly justify the choice of the comparator.

	1	2	3	4	5	
subitem not at all important	○	○	◉	○	○	essential

Cancella selezione

Does your paper address subitem 2a-ii? *

Copy and paste relevant sections from the manuscript (include quotes in quotation marks "like this" to indicate direct quotes from your manuscript), or elaborate on this item by providing additional information not in the ms, or briefly explain why the item is not applicable/relevant for your study

'Recent reviews point to challenges including small effect sizes, publication bias, reliance on self-report outcomes, and insufficient control for nonspecific treatment effects such as placebo and demand characteristics [8,9,10,11]
Demand characteristics and placebo effects are particularly salient in digital mental health studies, including mindfulness research. Participants may overreport improvements due to perceived researcher expectations [12] or inflated beliefs about the intervention's efficacy [13,14], especially when control conditions lack structural and experiential equivalence [9]. A high-powered pre-registered multi-site study [6] addressed this concern by comparing SAM to an active control (story listening), but its control condition lacked key features of meditation itself that may be needed to effectively control for non-specific factors and fully isolate mindfulness-specific effects.
The present study addresses these methodological limitations by pilot testing and evaluating a fully decentralized, self-administered mindfulness (SAM) intervention compared to a structurally matched sham control. This study aimed to assess the feasibility and acceptability of delivering both interventions entirely remotely, while exploring preliminary effects on stress-related outcomes. The design incorporated three methodological innovations intended to improve future scalability and internal validity: 1) A sham intervention designed to match in structure, expectancy and credibility to the SAM intervention capable of disentangling potential mindfulness-specific effects from non-specific effects (e.g., expectancy, demand characteristics); 2) remote collection of Heart Rate Variability (HRV), an objective physiological biomarker of stress; and 3) full decentralization of all procedures via smartphone allowing unsupervised delivery across multiple platforms to maximize reach and minimize barriers to participation and potential sources of bias.'

2b) In INTRODUCTION: Specific objectives or hypotheses

Does your paper address CONSORT subitem 2b? *

Copy and paste relevant sections from the manuscript (include quotes in quotation marks "like this" to indicate direct quotes from your manuscript), or elaborate on this item by providing additional information not in the ms, or briefly explain why the item is not applicable/relevant for your study

'Feasibility and acceptability were evaluated to inform the design of future trials. Feasibility was assessed through recruitment and retention rates (documented via a CONSORT flow diagram), cross-platform delivery evaluation (Qualtrics, WhatsApp, CameraHRV), adherence to protocols (including outcome measurement completeness), and physiological data quality. To contextualize our benchmarks, we drew on evidence from a meta-analysis of smartphone app trials for depressive symptoms [15], which reported an average 26.2% dropout rate (73.8% retention) across 18 RCTs, rising to 47.8% (52.2% retention) after adjusting for publication bias. Notably, studies incorporating human feedback and in-app mood monitoring demonstrated lower attrition. Aligning with these findings, our trial integrated WhatsApp for real-time human support and the app 'Camera HRV' for physiological self-monitoring, strategies hypothesized to mitigate dropout. However, given the pilot nature of our study and the need to ensure rigor for subsequent efficacy trials, we adopted stricter feasibility criteria than typical app-based studies: Recruitment of n = 60, retention >80%, and ≥70% valid HRV/STAI-6 adherence. These targets exceed both the unadjusted and adjusted retention rates reported by Torous et al., [14] reflecting our emphasis on optimizing engagement in a small-scale pilot. Acceptability was assessed via a 17-item usability questionnaire, open-ended feedback, and measures of credibility and expectancy.

While acceptability thresholds were not predefined, we interpreted results using benchmarks derived from System Usability Scale (SUS) literature [15,16]. Specifically, Brooke's retrospective work [15] highlights that mean item scores ≥4/5 on Likert scales (e.g., "strongly agree") correspond to SUS scores >80, a threshold associated with "excellent" usability in digital tools. Lewis [16] further underscores that such scores, when coupled with low variability (SD ≤0.8), reflect strong consensus among users, a critical marker of acceptability in pilot interventions. These criteria align with mobile health studies that simplify SUS principles for feasibility-focused trials while retaining methodological rigor.

Our primary intervention hypothesis posited greater self-reported stress reduction in the mindfulness group versus sham controls. Secondary objectives explored (1) HRV as a physiological stress marker, (2) Ecological Momentary Assessments (EMA)-derived transient states (e.g., fatigue), and (3) moderation by baseline traits (FFMQ, resilience, neuroticism). Bayesian methods were employed to estimate effect sizes and uncertainty, prioritizing practical interpretation over dichotomous significance testing. By aligning our feasibility framework with evidence-based strategies to reduce attrition (e.g., human engagement tools) while setting ambitious retention targets, this pilot bridges gaps identified in prior app trials and lays a robust foundation for fully powered efficacy studies.
'

METHODS

3a) Description of trial design (such as parallel, factorial) including allocation ratio

Does your paper address CONSORT subitem 3a? *

Copy and paste relevant sections from the manuscript (include quotes in quotation marks "like this" to indicate direct quotes from your manuscript), or elaborate on this item by providing additional information not in the ms, or briefly explain why the item is not applicable/relevant for your study

All study materials (meditation scripts, analytic code, de-identified data) are hosted on OSF [17], and the protocol was pre-registered (ClinicalTrials.gov: NCT06765889). The design followed a randomized, double-blind, 2-arm parallel-group framework, conducted remotely over 4 days (1 day for consent, 3 days for study procedures).

3b) Important changes to methods after trial commencement (such as eligibility criteria), with reasons

Does your paper address CONSORT subitem 3b? *

Copy and paste relevant sections from the manuscript (include quotes in quotation marks "like this" to indicate direct quotes from your manuscript), or elaborate on this item by providing additional information not in the ms, or briefly explain why the item is not applicable/relevant for your study

We did not implement changed to methods after that the trial started.

3b-i) Bug fixes, Downtimes, Content Changes

Bug fixes, Downtimes, Content Changes: ehealth systems are often dynamic systems. A description of changes to methods therefore also includes important changes made on the intervention or comparator during the trial (e.g., major bug fixes or changes in the functionality or content) (5-iii) and other “unexpected events” that may have influenced study design such as staff changes, system failures/downtimes, etc. [2].

	1	2	3	4	5	
subitem not at all important	○	◉	○	○	○	essential

Cancella selezione

Does your paper address subitem 3b-i?

Copy and paste relevant sections from the manuscript (include quotes in quotation marks "like this" to indicate direct quotes from your manuscript), or elaborate on this item by providing additional information not in the ms, or briefly explain why the item is not applicable/relevant for your study

No Bug fixes, Downtimes or other issues of this kind were encountered.

4a) Eligibility criteria for participants

Does your paper address CONSORT subitem 4a? *

Copy and paste relevant sections from the manuscript (include quotes in quotation marks "like this" to indicate direct quotes from your manuscript), or elaborate on this item by providing additional information not in the ms, or briefly explain why the item is not applicable/relevant for your study

To qualify, individuals had to be at least 21 years old, proficient in English, and willing to comply with all study procedures. They must not have practiced meditation within the previous six months, used recreational drugs, or had uncorrected vision or hearing impairments. Participants were also required to have no history of mental illness, no diagnosed major neurological or psychiatric condition in the past six months, and no current or recent (within the past week) use of psychoactive medications, including antidepressants, anxiolytics, hypnotics, or stimulants. Furthermore, individuals with any current or prior affiliation with A*STAR research teams were excluded.
Before proceeding, participants were informed that the screening included questions designed to determine their eligibility and that honest responses were essential. The survey was configured to present each screening question individually and to terminate immediately if any exclusion criterion was met. This approach was adopted to reduce unnecessary exposure to sensitive questions and to maintain participant comfort and confidentiality.

4a-i) Computer / Internet literacy

Computer / Internet literacy is often an implicit “de facto” eligibility criterion - this should be explicitly clarified.

	1	2	3	4	5	
subitem not at all important	○	○	○	●	○	essential

Cancella selezione

Does your paper address subitem 4a-i?

Copy and paste relevant sections from the manuscript (include quotes in quotation marks "like this" to indicate direct quotes from your manuscript), or elaborate on this item by providing additional information not in the ms, or briefly explain why the item is not applicable/relevant for your study

'Given that the study procedures were conducted online, participants were also required to have access to the internet and sufficient computer literacy to complete the screening and study tasks'.

4a-ii) Open vs. closed, web-based vs. face-to-face assessments:

Open vs. closed, web-based vs. face-to-face assessments: Mention how participants were recruited (online vs. offline), e.g., from an open access website or from a clinic, and clarify if this was a purely web-based trial, or there were face-to-face components (as part of the intervention or for assessment), i.e., to what degree got the study team to know the participant. In online-only trials, clarify if participants were quasi-anonymous and whether having multiple identities was possible or whether technical or logistical measures (e.g., cookies, email confirmation, phone calls) were used to detect/prevent these.

	1	2	3	4	5	
subitem not at all important	○	○	◉	○	○	essential

Cancella selezione

Does your paper address subitem 4a-ii? *

Copy and paste relevant sections from the manuscript (include quotes in quotation marks "like this" to indicate direct quotes from your manuscript), or elaborate on this item by providing additional information not in the ms, or briefly explain why the item is not applicable/relevant for your study

'Participants were recruited via an online screening form that assessed eligibility based on several predefined criteria to ensure the safety of participants and the integrity of the research.'

4a-iii) Information giving during recruitment

Information given during recruitment. Specify how participants were briefed for recruitment and in the informed consent procedures (e.g., publish the informed consent documentation as appendix, see also item X26), as this information may have an effect on user self-selection, user expectation and may also bias results.

	1	2	3	4	5	
subitem not at all important	○	○	◉	○	○	essential

Cancella selezione

Does your paper address subitem 4a-iii?

Copy and paste relevant sections from the manuscript (include quotes in quotation marks "like this" to indicate direct quotes from your manuscript), or elaborate on this item by providing additional information not in the ms, or briefly explain why the item is not applicable/relevant for your study

'All participants provided informed consent online via a Qualtrics survey. ' and also: 'Eligible participants received a Qualtrics link to review the study protocol and consent form. Due to the study's minimal risk (no medication or incidental findings), consent was obtained remotely without a witness. Participants declining consent were excluded. Those consenting completed additional screening questions about neurological/psychiatric conditions and concomitant medication; failure to meet criteria resulted in automatic termination.'

4b) Settings and locations where the data were collected

Does your paper address CONSORT subitem 4b? *

Copy and paste relevant sections from the manuscript (include quotes in quotation marks "like this" to indicate direct quotes from your manuscript), or elaborate on this item by providing additional information not in the ms, or briefly explain why the item is not applicable/relevant for your study

'This was an entirely remote pilot study conducted through smartphones, with no in-person interactions involved'.

4b-i) Report if outcomes were (self-)assessed through online questionnaires

Clearly report if outcomes were (self-)assessed through online questionnaires (as common in web-based trials) or otherwise.

	1	2	3	4	5	
subitem not at all important	○	○	◉	○	○	essential

Cancella selezione

Does your paper address subitem 4b-i? *

Copy and paste relevant sections from the manuscript (include quotes in quotation marks "like this" to indicate direct quotes from your manuscript), or elaborate on this item by providing additional information not in the ms, or briefly explain why the item is not applicable/relevant for your study

'Participants repeated the same procedure on Days 1, 2 and 3:
1. Completing pre-intervention self-report assessments (STAI-6, EMA, HRV).
2. Listening to a different mindfulness or sham meditation track, depending on group allocation.
3. Sitting quietly for three minutes to minimize confounding variables in subsequent HRV assessments.
4. Completing post-intervention self-report assessments (STAI-6, EMA, HRV).
'

4b-ii) Report how institutional affiliations are displayed

Report how institutional affiliations are displayed to potential participants [on ehealth media], as affiliations with prestigious hospitals or universities may affect volunteer rates, use, and reactions with regards to an intervention.(Not a required item – describe only if this may bias results)

	1	2	3	4	5	
subitem not at all important	◉	○	○	○	○	essential

Cancella selezione

Does your paper address subitem 4b-ii?

Copy and paste relevant sections from the manuscript (include quotes in quotation marks "like this" to indicate direct quotes from your manuscript), or elaborate on this item by providing additional information not in the ms, or briefly explain why the item is not applicable/relevant for your study

This item does not apply to our study.

5) The interventions for each group with sufficient details to allow replication, including how and when they were actually administered

5-i) Mention names, credential, affiliations of the developers, sponsors, and owners

Mention names, credential, affiliations of the developers, sponsors, and owners [6] (if authors/evaluators are owners or developer of the software, this needs to be declared in a "Conflict of interest" section or mentioned elsewhere in the manuscript).

	1	2	3	4	5	
subitem not at all important	○	○	◉	○	○	essential

Cancella selezione

Does your paper address subitem 5-i?

Copy and paste relevant sections from the manuscript (include quotes in quotation marks "like this" to indicate direct quotes from your manuscript), or elaborate on this item by providing additional information not in the ms, or briefly explain why the item is not applicable/relevant for your study

'Mindfulness Intervention. The mindfulness tracks were written and recorded by a certified MBSR instructor from Singapore with over a decade of experience. The intervention included three unique guided practices, each designed to strengthen attentional stability and meta-awareness, key mechanisms associated with effective mindfulness training and stress regulation [31,32,33]. The sessions focused on the following elements:

1. Attentional Stability: Participants were guided to anchor their focus on present-moment bodily experiences, such as breath sensations, physical posture, or environmental stimuli, to enhance their ability to sustain attention without distraction.
2. Mindful Meta-Awareness: The practice encouraged nonreactive and nonjudgmental observation of thoughts, emotions, and bodily sensations, fostering acceptance and self-regulation skills.

Each track emphasized present-moment awareness using different modalities:

- Day 1: Formal mindfulness practice (i.e., body awareness meditation): Encouraged attentiveness to bodily sensations, surrounding sounds, and breath, training participants to maintain awareness despite distractions.
- Day 2: Informal mindfulness practice (i.e., mindful brushing of teeth): Participants were guided to integrate mindfulness into the act of brushing their teeth, approaching this habitual behaviour with curiosity and full attentiveness. The practice encourages awareness of sensations, movements, and breath, fostering a mindful presence.
- Day 3: Breath awareness practice: Focused on observing the natural rhythm of breathing without trying to change it, reinforcing nonjudgmental awareness.

Sham Meditation Condition. The first and second authors developed the sham meditation condition, with the second author contributing expertise in implementing such control conditions [8]. After finalizing the sham, it was reviewed by the mindfulness instructor to ensure no core mindfulness components remained. Designed to structurally mirror the mindfulness intervention, the sham condition intentionally excluded key mindfulness mechanisms (e.g., attentional stability, meta-awareness). The goal was to create an experience that matched the mindfulness group in duration, delivery format, and voice, thereby controlling for non-specific factors, while guiding participants through exercises that emphasized multitasking and free-flowing thought, creating an experience that felt meditative to novices without delivering active mindfulness training [8].

1. No Attentional Stability: Unlike the mindfulness condition, the sham sessions did not provide an anchor for attention. Instead, participants were encouraged to allow their thoughts to wander freely, shifting between different ideas and tasks.
2. No Meta-Cognitive Guidance: Instructions omitted any emphasis on nonjudgmental awareness, acceptance, or self-observation. Instead, participants were prompted to engage with multiple thoughts at once, emphasizing cognitive agility and rapid task-switching.

Each sham session included:

- Day 1: Associative Thinking (e.g., encouraging free-association thinking): Participants were guided to let their minds jump between thoughts, focusing on productivity and efficiency rather than present-moment awareness.
- Day 2: Routine Task Engagement (i.e., brushing teeth while thinking about tasks): Participants were encouraged to perform everyday activities (e.g., brushing teeth) while reflecting on unrelated tasks. This approach emphasized multitasking without cultivating mindful curiosity or awareness.
- Day 3: Creative Exploration (e.g., picking a thought and expanding on it): Participants were instructed to select a thought and expand on it through imaginative thinking. This exercise promoted mental flexibility and creativity but did not involve sustained focus on present experiences.

The script of the active and control condition with the corresponding audio tracks can be found in our OSF page of the project [17]'

5-ii) Describe the history/development process

Describe the history/development process of the application and previous formative evaluations (e.g., focus groups, usability testing), as these will have an impact on adoption/use rates and help with interpreting results.

	1	2	3	4	5	
subitem not at all important	○	◉	○	○	○	essential

Cancella selezione

Does your paper address subitem 5-ii?

Copy and paste relevant sections from the manuscript (include quotes in quotation marks "like this" to indicate direct quotes from your manuscript), or elaborate on this item by providing additional information not in the ms, or briefly explain why the item is not applicable/relevant for your study

'Intervention Development
To ensure the mindfulness and sham interventions were grounded in best practices and optimized for a brief, decentralized format, we conducted a formative focus group with five certified international Mindfulness-Based Stress Reduction (MBSR) instructors prior to the pilot trial. The primary objective was to gather expert consensus on the core components, duration, and structure of a mindfulness protocol suitable for a multi-day, self-administered study with a non-clinical population experiencing stress. The expert panel's recommendations directly shaped the core parameters of the final study design. To ensure accessibility for beginners in a remote context, the instructors guided us to focus on foundational practices, leading to the selection of exercises centered on attentional stability through breath awareness and mindful meta-awareness of thoughts and sensations.
The panel also advised that a brief daily session of approximately 10-12 minutes would be optimal for maximizing engagement and adherence. Based on this, we implemented a three-day "taster" protocol, which was considered an appropriate duration to robustly test the feasibility and acceptability of the intervention before scaling to a longer trial. The design of the sham condition was also informed by this focus group, which highlighted the challenge of creating a credible control. The experts suggested leveraging common misconceptions about meditation (e.g., that it is purely a relaxation or thought-engagement exercise), which led to our final design emphasizing free-flowing thought and multitasking rather than present-moment awareness. The summary and the transcript of the focus group can be found in the OSF page of the project [17].
'

5-iii) Revisions and updating

Revisions and updating. Clearly mention the date and/or version number of the application/intervention (and comparator, if applicable) evaluated, or describe whether the intervention underwent major changes during the evaluation process, or whether the development and/or content was "frozen" during the trial. Describe dynamic components such as news feeds or changing content which may have an impact on the replicability of the intervention (for unexpected events see item 3b).

	1	2	3	4	5	
subitem not at all important	○	◉	○	○	○	essential

Cancella selezione

Does your paper address subitem 5-iii?

Copy and paste relevant sections from the manuscript (include quotes in quotation marks "like this" to indicate direct quotes from your manuscript), or elaborate on this item by providing additional information not in the ms, or briefly explain why the item is not applicable/relevant for your study

The mindfulness intervention was fully developed by the mindfulness instructor, while the sham was revised by the team as stated: 'Sham Meditation Condition. The first and second authors developed the sham meditation condition, with the second author contributing expertise in implementing such control conditions [8]. After finalizing the sham, it was reviewed by the mindfulness instructor to ensure no core mindfulness components remained. '

5-iv) Quality assurance methods

Provide information on quality assurance methods to ensure accuracy and quality of information provided [1], if applicable.

	1	2	3	4	5	
subitem not at all important	○	○	◉	○	○	essential

Cancella selezione

Does your paper address subitem 5-iv?

Copy and paste relevant sections from the manuscript (include quotes in quotation marks "like this" to indicate direct quotes from your manuscript), or elaborate on this item by providing additional information not in the ms, or briefly explain why the item is not applicable/relevant for your study

For the mindfulness intervention: 'Mindfulness Intervention. The mindfulness tracks were written and recorded by a certified MBSR instructor from Singapore with over a decade of experience. The intervention included three unique guided practices, each designed to strengthen attentional stability and meta-awareness, key mechanisms associated with effective mindfulness training and stress regulation [31,32,33]. ' For the control condition: 'Sham Meditation Condition. The first and second authors developed the sham meditation condition, with the second author contributing expertise in implementing such control conditions [8]. After finalizing the sham, it was reviewed by the mindfulness instructor to ensure no core mindfulness components remained. Designed to structurally mirror the mindfulness intervention, the sham condition intentionally excluded key mindfulness mechanisms (e.g., attentional stability, meta-awareness). '

5-v) Ensure replicability by publishing the source code, and/or providing screenshots/screen-capture video, and/or providing flowcharts of the algorithms used

Ensure replicability by publishing the source code, and/or providing screenshots/screen-capture video, and/or providing flowcharts of the algorithms used. Replicability (i.e., other researchers should in principle be able to replicate the study) is a hallmark of scientific reporting.

	1	2	3	4	5	
subitem not at all important	○	○	○	○	◉	essential

Cancella selezione

Does your paper address subitem 5-v?

Copy and paste relevant sections from the manuscript (include quotes in quotation marks "like this" to indicate direct quotes from your manuscript), or elaborate on this item by providing additional information not in the ms, or briefly explain why the item is not applicable/relevant for your study

'All study materials (meditation scripts, analytic code, de-identified data) are hosted on OSF [17], and the protocol was pre-registered (ClinicalTrials.gov: NCT06765889). The design followed a randomized, double-blind, 2-arm parallel-group framework, conducted remotely over 4 days (1 day for consent, 3 days for study procedures). This was an entirely remote pilot study conducted through smartphones, with no in-person interactions involved'

5-vi) Digital preservation

Digital preservation: Provide the URL of the application, but as the intervention is likely to change or disappear over the course of the years; also make sure the intervention is archived (Internet Archive, webcitation.org, and/or publishing the source code or screenshots/videos alongside the article). As pages behind login screens cannot be archived, consider creating demo pages which are accessible without login.

	1	2	3	4	5	
subitem not at all important	○	◉	○	○	○	essential

Cancella selezione

Does your paper address subitem 5-vi?

Copy and paste relevant sections from the manuscript (include quotes in quotation marks "like this" to indicate direct quotes from your manuscript), or elaborate on this item by providing additional information not in the ms, or briefly explain why the item is not applicable/relevant for your study

'All study materials (meditation scripts, analytic code, de-identified data) are hosted on OSF [17], and the protocol was pre-registered (ClinicalTrials.gov: NCT06765889). The design followed a randomized, double-blind, 2-arm parallel-group framework, conducted remotely over 4 days (1 day for consent, 3 days for study procedures). This was an entirely remote pilot study conducted through smartphones, with no in-person interactions involved.'

5-vii) Access

Access: Describe how participants accessed the application, in what setting/context, if they had to pay (or were paid) or not, whether they had to be a member of specific group. If known, describe how participants obtained “access to the platform and Internet” [1]. To ensure access for editors/reviewers/readers, consider to provide a “backdoor” login account or demo mode for reviewers/readers to explore the application (also important for archiving purposes, see vi).

	1	2	3	4	5	
subitem not at all important	○	○	◉	○	○	essential

Cancella selezione

Does your paper address subitem 5-vii? *

Copy and paste relevant sections from the manuscript (include quotes in quotation marks "like this" to indicate direct quotes from your manuscript), or elaborate on this item by providing additional information not in the ms, or briefly explain why the item is not applicable/relevant for your study

After consent, participants were aggregated into cohorts of 20 participants and notified that the study would commence within two weeks. Cohorts were initiated weekly, with additional cohorts added as needed to reach the recruiting target of 60 participants. Following Whitehead and colleagues’ [18] calculations for continuous outcomes, our sample size (n = 60) provided adequate precision to estimate feasibility parameters. Eligible participants received the following via a WhatsApp message from the study coordinator:

1. A Qualtrics survey link with a unique anonymous access code.
2. Instructions for downloading and using the ‘Camera HRV’ app (Altini, 2019), a third-party smartphone application that measures Heart Rate Variability (HRV) via photoplethysmography (PPG) without requiring external hardware. Guidelines included: a) Proper measurement protocols, b) Steps to submit recorded data as a .csv file, c) A free download code for the ‘Camera HRV’ app.

HRV data were collected via the app, and participants submitted files through WhatsApp to the study coordinator.

5-viii) Mode of delivery, features/functionalities/components of the intervention and comparator, and the theoretical framework

Describe mode of delivery, features/functionalities/components of the intervention and comparator, and the theoretical framework [6] used to design them (instructional strategy [1], behaviour change techniques, persuasive features, etc., see e.g., [7, 8] for terminology). This includes an in-depth description of the content (including where it is coming from and who developed it) [1]," whether [and how] it is tailored to individual circumstances and allows users to track their progress and receive feedback" [6]. This also includes a description of communication delivery channels and – if computer-mediated communication is a component – whether communication was synchronous or asynchronous [6]. It also includes information on presentation strategies [1], including page design principles, average amount of text on pages, presence of hyperlinks to other resources, etc. [1].

	1	2	3	4	5	
subitem not at all important	○	○	◉	○	○	essential

Cancella selezione

Does your paper address subitem 5-viii? *

Copy and paste relevant sections from the manuscript (include quotes in quotation marks "like this" to indicate direct quotes from your manuscript), or elaborate on this item by providing additional information not in the ms, or briefly explain why the item is not applicable/relevant for your study

The design followed a randomized, double-blind, 2-arm parallel-group framework, conducted remotely over 4 days. This was an entirely remote pilot study conducted through smartphones, with no in-person interactions involved.
To ensure the mindfulness and sham interventions were grounded in best practices, we conducted a formative focus group with five certified international Mindfulness-Based Stress Reduction (MBSR) instructors prior to the pilot trial. The panel's recommendations directly shaped the core parameters of the study design, including session duration (10-12 minutes) and the focus on foundational practices for beginners. The design of the sham condition was also informed by this focus group, leveraging common misconceptions about meditation to create a credible control.
The mindfulness tracks were written and recorded by a certified MBSR instructor. The intervention included three unique guided practices designed to strengthen attentional stability and meta-awareness. The daily sessions included: (1) body awareness meditation, (2) mindful brushing of teeth, and (3) breath awareness practice.
The sham meditation condition was developed by the first and second authors and reviewed by the mindfulness instructor. It was designed to structurally mirror the mindfulness intervention in duration and delivery format but intentionally excluded key mechanisms. It guided participants through exercises emphasizing multitasking and free-flowing thought, such as: (1) associative thinking, (2) routine task engagement while thinking about other tasks, and (3) creative exploration of a thought.
Both conditions were delivered via audio tracks embedded in a Qualtrics survey, which could not be skipped for 10 minutes. Communication with participants was conducted asynchronously via WhatsApp for delivering survey links and receiving data files.
The intervention was not tailored to individual circumstances, and the system did not provide direct feedback on performance. Participants tracked their state via daily pre- and post-intervention assessments (STAI-6, EMA) and physiological responses (HRV).
The script of the active and control condition with the corresponding audio tracks can be found in our OSF page of the project [17].

5-ix) Describe use parameters

Describe use parameters (e.g., intended “doses” and optimal timing for use). Clarify what instructions or recommendations were given to the user, e.g., regarding timing, frequency, heaviness of use, if any, or was the intervention used ad libitum.

	1	2	3	4	5	
subitem not at all important	○	○	◉	○	○	essential

Cancella selezione

Does your paper address subitem 5-ix?

Copy and paste relevant sections from the manuscript (include quotes in quotation marks "like this" to indicate direct quotes from your manuscript), or elaborate on this item by providing additional information not in the ms, or briefly explain why the item is not applicable/relevant for your study

'Participants were instructed to complete their daily sessions at approximately the same time each day to ensure consistency. They were informed that the sessions on Days 1 and 3 would each take approximately 35 minutes, while the Day 2 session would take around 25 minutes.'

5-x) Clarify the level of human involvement

Clarify the level of human involvement (care providers or health professionals, also technical assistance) in the e-intervention or as co-intervention (detail number and expertise of professionals involved, if any, as well as “type of assistance offered, the timing and frequency of the support, how it is initiated, and the medium by which the assistance is delivered”. It may be necessary to distinguish between the level of human involvement required for the trial, and the level of human involvement required for a routine application outside of a RCT setting (discuss under item 21 – generalizability).

	1	2	3	4	5	
subitem not at all important	○	○	◉	○	○	essential

Cancella selezione

Does your paper address subitem 5-x?

Copy and paste relevant sections from the manuscript (include quotes in quotation marks "like this" to indicate direct quotes from your manuscript), or elaborate on this item by providing additional information not in the ms, or briefly explain why the item is not applicable/relevant for your study

Human involvement was minimal and for administrative purposes only, with no clinical, therapeutic, or technical support provided by the research team.
A study coordinator was the sole point of contact. The coordinator's involvement included:
Screening: Flagging duplicate submissions during recruitment.
Initiating the study: Sending a one-time message via WhatsApp at the start of the study containing the Qualtrics survey link and instructions for the HRV application.
Data Collection: Receiving the .csv data files submitted by participants via WhatsApp at the end of the study.
Concluding the study: Managing compensation for participants.
All communication was asynchronous and conducted via WhatsApp. The intervention itself was entirely self-administered by the participants.

5-xi) Report any prompts/reminders used

Report any prompts/reminders used: Clarify if there were prompts (letters, emails, phone calls, SMS) to use the application, what triggered them, frequency etc. It may be necessary to distinguish between the level of prompts/reminders required for the trial, and the level of prompts/reminders for a routine application outside of a RCT setting (discuss under item 21 – generalizability).

	1	2	3	4	5	
subitem not at all important	○	○	◉	○	○	essential

Cancella selezione

Does your paper address subitem 5-xi? *

Copy and paste relevant sections from the manuscript (include quotes in quotation marks "like this" to indicate direct quotes from your manuscript), or elaborate on this item by providing additional information not in the ms, or briefly explain why the item is not applicable/relevant for your study

No prompts or reminders were used to encourage daily use of the intervention. After receiving the initial instructions and survey link via WhatsApp, participants were expected to independently follow the daily procedure for the three consecutive days of the study.

5-xii) Describe any co-interventions (incl. training/support)

Describe any co-interventions (incl. training/support): Clearly state any interventions that are provided in addition to the targeted eHealth intervention, as ehealth intervention may not be designed as stand-alone intervention. This includes training sessions and support [1]. It may be necessary to distinguish between the level of training required for the trial, and the level of training for a routine application outside of a RCT setting (discuss under item 21 – generalizability.

	1	2	3	4	5	
subitem not at all important	(●)	○	○	○	○	essential

Cancella selezione

Does your paper address subitem 5-xii? *

Copy and paste relevant sections from the manuscript (include quotes in quotation marks "like this" to indicate direct quotes from your manuscript), or elaborate on this item by providing additional information not in the ms, or briefly explain why the item is not applicable/relevant for your study

This does not apply to this study.

6a) Completely defined pre-specified primary and secondary outcome measures, including how and when they were assessed

Does your paper address CONSORT subitem 6a? *

Copy and paste relevant sections from the manuscript (include quotes in quotation marks "like this" to indicate direct quotes from your manuscript), or elaborate on this item by providing additional information not in the ms, or briefly explain why the item is not applicable/relevant for your study

Primary Outcomes:

The primary outcomes were feasibility, acceptability, and changes in self-reported stress (STAI-6).
"Primary Outcomes: Feasibility, Acceptability and STAI-6 We first evaluated feasibility through three key metrics: (1) Recruitment yield (completed/enrolled ratio), (2) retention rates, and (3) protocol adherence (STAI-6/HRV completion percentages)."
"Acceptability was assessed via the 17-item usability questionnaire (analysed descriptively) and qualitative coding of open-ended responses." This was assessed at the end of the study on Day 3.
"The following scales were administered twice daily—once before and once after listening to each audio track: State Trait Anxiety Inventory – Short Form (STAI-6): The STAI-6 is a validated short-form version of the 40-item State-Trait Anxiety Inventory. It consists of six items measuring current anxiety symptoms “right now” using a 4-point Likert scale (α = .88 (95% CI [.86, .90])."

Secondary Outcomes:
The secondary outcomes were changes in Heart Rate Variability (HRV) and Ecological Momentary Assessments (EMAs), as well as the moderating effects of several individual difference measures.
"Secondary Outcomes: Intervention Effects We repeated the same Bayesian latent growth curve modelling approach to assess the effectiveness of Heart Rate Variability (HRV) and the Ecological Momentary Assessments (EMAs) we measured."
These were assessed twice daily, pre- and post-intervention:
"Ecological Momentary Assessment (EMA): Participants rated their current state using a slider scale from 1 (not at all) to 100 (very much). The following 7 questions were used to assess various psychological and physical states..."
"Heart Rate Variability: Heart Rate Variability (HRV) was measured to assess parasympathetic activation using the photoplethysmography (PPG)-based ‘Camera HRV’ app..."
The manuscript also pre-specified the assessment of moderators as a secondary analysis:
"Furthermore, we tested whether FFMQ (Five Facet Mindfulness Questionnaire), Neuroticism, and Resilience had any impact on the self-reported stress reduction effect of the mindfulness condition versus sham. To do so, we incorporated these individual difference measures as covariates in the model to examine their moderating effects..."
These moderators were assessed once at the beginning of the study:
"They then completed demographic questions... and the following scales: Neuroticism Subscale of the IPIP (IPIP-20)... Resilience Scale 14 (RS-14)... Five Facet Mindfulness Questionnaire – Short Form (FFMQ-SF)..."

6a-i) Online questionnaires: describe if they were validated for online use and apply CHERRIES items to describe how the questionnaires were designed/deployed

If outcomes were obtained through online questionnaires, describe if they were validated for online use and apply CHERRIES items to describe how the questionnaires were designed/deployed [9].

	1	2	3	4	5	
subitem not at all important	○	○	◉	○	○	essential

Cancella selezione

Does your paper address subitem 6a-i?

Copy and paste relevant sections from manuscript text

The following sections describe the design and deployment of the online questionnaires, which were administered using Qualtrics:

Ethical Approval and Consent:
"This study was conducted in accordance with the Declaration of Helsinki and approved by the A*STAR Institutional Review Board on November 26th 2023 (ASTAR IRB Reference: 2024-102). All participants provided informed consent online via a Qualtrics survey... Eligible participants received a Qualtrics link to review the study protocol and consent form. Due to the study's minimal risk (no medication or incidental findings), consent was obtained remotely without a witness. Participants declining consent were excluded."

Survey Design, Data Quality and Prevention of Duplicate Submissions:
"Participants were recruited via an online screening form... Before proceeding, participants were informed that the screening included questions designed to determine their eligibility and that honest responses were essential. The survey was configured to present each screening question individually and to terminate immediately if any exclusion criterion was met. This approach was adopted to reduce unnecessary exposure to sensitive questions and to maintain participant comfort and confidentiality."

"Phone numbers were collected to prevent duplicate submissions... Duplicates were flagged by the study coordinator, and ineligible participants' data were immediately discarded."

"Eligible participants received the following via a WhatsApp message from the study coordinator: 1. A Qualtrics survey link with a unique anonymous access code."

6a-ii) Describe whether and how “use” (including intensity of use/dosage) was defined/measured/monitored

Describe whether and how “use” (including intensity of use/dosage) was defined/measured/monitored (logins, logfile analysis, etc.). Use/adoption metrics are important process outcomes that should be reported in any ehealth trial.

	1	2	3	4	5	
subitem not at all important	○	○	○	○	○	essential

Does your paper address subitem 6a-ii?

Copy and paste relevant sections from manuscript text

The "dosage" of the intervention was standardized and its use was enforced by the study platform. This is described in the Randomization and Experimental Conditions section:

"The sessions were embedded within the Qualtrics survey and could not be skipped for 10 minutes (matching the time of the audio track)."

Furthermore, "use" was operationalized as "protocol adherence" and was measured as a primary outcome by tracking the completion of daily tasks. This is described in the Statistical Analysis section:

"Primary Outcomes: Feasibility, Acceptability and STAI-6
We first evaluated feasibility through three key metrics: (1) Recruitment yield (completed/enrolled ratio), (2) retention rates, and (3) protocol adherence (STAI-6/HRV completion percentages)."

6a-iii) Describe whether, how, and when qualitative feedback from participants was obtained

Describe whether, how, and when qualitative feedback from participants was obtained (e.g., through emails, feedback forms, interviews, focus groups).

	1	2	3	4	5	
subitem not at all important	○	◉	○	○	○	essential

Cancella selezione

Does your paper address subitem 6a-iii?

Copy and paste relevant sections from manuscript text

The manuscript describes that qualitative feedback was obtained at the end of the study using open-ended questions within a final questionnaire.

This is detailed in the End of Study Assessments section, under the subsection for the "Usability and Acceptability Questionnaire":

"Open-ended questions (e.g., challenges, time commitment) expanded on quantitative data, following recommendations for mixed-methods usability assessments [28]."

6b) Any changes to trial outcomes after the trial commenced, with reasons

Does your paper address CONSORT subitem 6b? *

Copy and paste relevant sections from the manuscript (include quotes in quotation marks "like this" to indicate direct quotes from your manuscript), or elaborate on this item by providing additional information not in the ms, or briefly explain why the item is not applicable/relevant for your study

There were no changes to trial outcomes after the trial started.

7a) How sample size was determined

NPT: When applicable, details of whether and how the clustering by care provides or centers was addressed

7a-i) Describe whether and how expected attrition was taken into account when calculating the sample size

Describe whether and how expected attrition was taken into account when calculating the sample size.

	1	2	3	4	5	
subitem not at all important	○	○	○	○	◉	essential

Cancella selezione

Does your paper address subitem 7a-i?

Copy and paste relevant sections from manuscript title (include quotes in quotation marks "like this" to indicate direct quotes from your manuscript), or elaborate on this item by providing additional information not in the ms, or briefly explain why the item is not applicable/relevant for your study

"Following Whitehead and colleagues' [18] calculations for continuous outcomes, our sample size (n = 60) provided adequate precision to estimate feasibility parameters."

7b) When applicable, explanation of any interim analyses and stopping guidelines

Does your paper address CONSORT subitem 7b? *

Copy and paste relevant sections from the manuscript (include quotes in quotation marks "like this" to indicate direct quotes from your manuscript), or elaborate on this item by providing additional information not in the ms, or briefly explain why the item is not applicable/relevant for your study

This item is not applicable. The study protocol did not include any planned interim analyses or formal stopping guidelines.

8a) Method used to generate the random allocation sequence

NPT: When applicable, how care providers were allocated to each trial group

Does your paper address CONSORT subitem 8a? *

Copy and paste relevant sections from the manuscript (include quotes in quotation marks "like this" to indicate direct quotes from your manuscript), or elaborate on this item by providing additional information not in the ms, or briefly explain why the item is not applicable/relevant for your study

"Participants were randomly assigned via Qualtrics to one of two experimental conditions: A mindfulness intervention or a sham meditation condition. The randomization process was implemented using the built-in Randomizer feature within the Qualtrics Survey Flow. This tool was configured to assign participants to the experimental conditions in a 1:1 ratio by using the 'evenly present elements' option, ensuring a balanced allocation across the duration of the study."

8b) Type of randomisation; details of any restriction (such as blocking and block size)

Does your paper address CONSORT subitem 8b? *

Copy and paste relevant sections from the manuscript (include quotes in quotation marks "like this" to indicate direct quotes from your manuscript), or elaborate on this item by providing additional information not in the ms, or briefly explain why the item is not applicable/relevant for your study

The randomization type was managed by the survey software. The randomization process was restricted using the built-in ‘evenly present elements’ option within the Qualtrics Survey Flow. This feature functions as a form of restricted randomization by ensuring a balanced allocation in a 1:1 ratio across the two conditions throughout the study's duration. A specific block size was not pre-defined, as the software dynamically maintains balance as participants are enrolled.

9) Mechanism used to implement the random allocation sequence (such as sequentially numbered containers), describing any steps taken to conceal the sequence until interventions were assigned

Does your paper address CONSORT subitem 9? *

Copy and paste relevant sections from the manuscript (include quotes in quotation marks "like this" to indicate direct quotes from your manuscript), or elaborate on this item by providing additional information not in the ms, or briefly explain why the item is not applicable/relevant for your study

"The random allocation sequence was implemented automatically using the built-in Randomizer feature within the Qualtrics Survey Flow. This software-based mechanism concealed the allocation sequence from both participants and the research team until the moment of assignment. As each participant progressed through the survey, the platform assigned them to a condition in real-time, making it impossible to foresee the allocation and ensuring concealment was maintained."

10) Who generated the random allocation sequence, who enrolled participants, and who assigned participants to interventions

Does your paper address CONSORT subitem 10? *

Copy and paste relevant sections from the manuscript (include quotes in quotation marks "like this" to indicate direct quotes from your manuscript), or elaborate on this item by providing additional information not in the ms, or briefly explain why the item is not applicable/relevant for your study

1) Who generated the random allocation sequence: The sequence was generated automatically by the survey software, as stated in the Randomization and Experimental Conditions section: "The randomization process was implemented using the built-in Randomizer feature within the Qualtrics Survey Flow."
2) Who enrolled participants: The study coordinator enrolled participants. This is described in the Recruitment and Eligibility and Procedure sections, where the "study coordinator" is mentioned flagging duplicates and sending the survey link to eligible participants.
3) Who assigned participants to interventions: The Qualtrics software platform assigned participants to their intervention group automatically and in real-time as they progressed through the survey.

11a) If done, who was blinded after assignment to interventions (for example, participants, care providers, those assessing outcomes) and how

NPT: Whether or not administering co-interventions were blinded to group assignment

11a-i) Specify who was blinded, and who wasn’t

Specify who was blinded, and who wasn’t. Usually, in web-based trials it is not possible to blind the participants [1, 3] (this should be clearly acknowledged), but it may be possible to blind outcome assessors, those doing data analysis or those administering co-interventions (if any).

	1	2	3	4	5	
subitem not at all important	○	○	◉	○	○	essential

Cancella selezione

Does your paper address subitem 11a-i? *

Copy and paste relevant sections from the manuscript (include quotes in quotation marks "like this" to indicate direct quotes from your manuscript), or elaborate on this item by providing additional information not in the ms, or briefly explain why the item is not applicable/relevant for your study

This was a double-blind trial.

Participants were blinded to their group assignment. This was achieved by designing a sham condition that was structurally equivalent to the active intervention.

"The design followed a randomized, double-blind, 2-arm parallel-group framework..."

"Designed to structurally mirror the mindfulness intervention, the sham condition intentionally excluded key mindfulness mechanisms... The goal was to create an experience that matched the mindfulness group in duration, delivery format, and voice, thereby controlling for non-specific factors, while guiding participants through exercises that emphasized multitasking and free-flowing thought, creating an experience that felt meditative to novices..."

Blinding of Those Assessing Outcomes:
The primary and secondary outcomes were self-reported by participants through automated Qualtrics questionnaires or collected via a third-party application ('Camera HRV'). The study coordinator who received the final data files was not involved in the intervention and was unaware of participant group allocation.

Assessment of Blinding Success:
The success of participant blinding was formally assessed at the end of the study using two methods:
Perceived Awareness of the Research Hypothesis (PARH): "The PARH is a four-item quantitative self-report tool designed to assess the potential impact of demand characteristics in research settings."
Expectancy and Credibility Assessment: "To evaluate credibility, participants responded to a two-part question: 'If you were informed that you might have received either meditation training or control training, which type do you believe you received? (Meditation, Control).' [and] 'How confident are you in your answer? (0 = Not at all confident, 10 = Extremely confident).'"

11a-ii) Discuss e.g., whether participants knew which intervention was the "intervention of interest" and which one was the "comparator"

Informed consent procedures (4a-ii) can create biases and certain expectations - discuss e.g., whether participants knew which intervention was the "intervention of interest" and which one was the "comparator".

	1	2	3	4	5	
subitem not at all important	○	○	◉	○	○	essential

Cancella selezione

Does your paper address subitem 11a-ii?

Copy and paste relevant sections from the manuscript (include quotes in quotation marks "like this" to indicate direct quotes from your manuscript), or elaborate on this item by providing additional information not in the ms, or briefly explain why the item is not applicable/relevant for your study

"The study formally assessed participant awareness and expectations using two specific measures at the end of the trial:

Perceived Awareness of the Research Hypothesis (PARH): "The PARH is a four-item quantitative self-report tool designed to assess the potential impact of demand characteristics in research settings. Participants responded to statements using a 7-point Likert-type scale... Example statements included: "I was aware of the researchers' objectives in this study." or "I was uncertain about the researchers' intentions in conducting this research."

Expectancy and Credibility of the Intervention: "To evaluate credibility, participants responded to a two-part question: 'If you were informed that you might have received either meditation training or control training, which type do you believe you received? (Meditation, Control).' [and] 'How confident are you in your answer? (0 = Not at all confident, 10 = Extremely confident).'"

11b) If relevant, description of the similarity of interventions

(this item is usually not relevant for ehealth trials as it refers to similarity of a placebo or sham intervention to a active medication/intervention)

Does your paper address CONSORT subitem 11b? *

Copy and paste relevant sections from the manuscript (include quotes in quotation marks "like this" to indicate direct quotes from your manuscript), or elaborate on this item by providing additional information not in the ms, or briefly explain why the item is not applicable/relevant for your study

A key feature of the trial was ensuring the sham condition was as similar as possible to the active mindfulness intervention in all aspects except for the core therapeutic mechanisms.
The interventions were matched on the following structural features:
Delivery Format and Voice: Both conditions consisted of audio-guided sessions delivered via Qualtrics and were recorded by the same certified MBSR instructor to maintain structural equivalence.
Duration: Both the mindfulness and sham sessions were 10 minutes long.
This is detailed in the Sham Meditation Condition section: 'Designed to structurally mirror the mindfulness intervention, the sham condition intentionally excluded key mindfulness mechanisms (e.g., attentional stability, meta-awareness). The goal was to create an experience that matched the mindfulness group in duration, delivery format, and voice, thereby controlling for non-specific factors..."'

12a) Statistical methods used to compare groups for primary and secondary outcomes

NPT: When applicable, details of whether and how the clustering by care providers or centers was addressed

Does your paper address CONSORT subitem 12a? *

Copy and paste relevant sections from the manuscript (include quotes in quotation marks "like this" to indicate direct quotes from your manuscript), or elaborate on this item by providing additional information not in the ms, or briefly explain why the item is not applicable/relevant for your study

For Primary Outcomes (Feasibility, Acceptability, STAI-6):

"We first evaluated feasibility through three key metrics: (1) Recruitment yield (completed/enrolled ratio), (2) retention rates, and (3) protocol adherence (STAI-6/HRV completion percentages). Acceptability was assessed via the 17-item usability questionnaire (analysed descriptively) and qualitative coding of open-ended responses.

We implemented a Bayesian latent growth curve model using the 'brms' package [34] to examine the effects of the mindfulness intervention on self-reported stress (measured via STAI-6). We specified a model that included: Fixed effects for the time point of the assessment (pre vs. post), the condition (Mindfulness vs. Sham), and their interaction. A covariate for day to account for potential daily fluctuations. Random slopes for time point that vary by participant to account for individual differences in responsiveness to the intervention.

We used weakly informative priors: Normal (0, 1) for fixed effects, normal (0, 5) for the intercept, Cauchy (0, 2) for random effects SDs... To evaluate the evidence for the experimental manipulation, we compared the full model (including the time point × condition interaction) to a null model without this interaction term. We used a Bayes Factor (BF10) comparison between the full and null models to quantify the relative evidence for each model."

For Secondary Outcomes:

"We repeated the same Bayesian latent growth curve modelling approach to assess the effectiveness of Heart Rate Variability (HRV) and the Ecological Momentary Assessments (EMAs) we measured. Furthermore, we tested whether FFMQ (Five Facet Mindfulness Questionnaire), Neuroticism, and Resilience had any impact on the self-reported stress reduction effect... by adding interaction terms between condition and each moderator... For each analysis, the full model (including the interaction term) was compared to a corresponding reduced model (excluding the interaction) using Bayes Factors to quantify the evidence for a moderating effect."

12a-i) Imputation techniques to deal with attrition / missing values

Imputation techniques to deal with attrition / missing values: Not all participants will use the intervention/comparator as intended and attrition is typically high in ehealth trials. Specify how participants who did not use the application or dropped out from the trial were treated in the statistical analysis (a complete case analysis is strongly discouraged, and simple imputation techniques such as LOCF may also be problematic [4]).

	1	2	3	4	5	
subitem not at all important	○	○	○	◉	○	essential

Cancella selezione

Does your paper address subitem 12a-i? *

Copy and paste relevant sections from the manuscript (include quotes in quotation marks "like this" to indicate direct quotes from your manuscript), or elaborate on this item by providing additional information not in the ms, or briefly explain why the item is not applicable/relevant for your study

The statistical approach chosen, a Bayesian latent growth curve model, inherently handles missing data from participant attrition without requiring traditional imputation techniques. By using a full information approach, the model leverages all available data points from every participant, even from those who dropped out, to estimate the growth trajectories. This method is superior to complete-case analysis as it avoids bias and loss of statistical power associated with high attrition rates common in e-health trials.

12b) Methods for additional analyses, such as subgroup analyses and adjusted analyses

Does your paper address CONSORT subitem 12b? *

Copy and paste relevant sections from the manuscript (include quotes in quotation marks "like this" to indicate direct quotes from your manuscript), or elaborate on this item by providing additional information not in the ms, or briefly explain why the item is not applicable/relevant for your study

"Methods for additional analyses were pre-specified. To investigate potential moderators of the intervention effect, we conducted adjusted analyses incorporating individual difference measures as covariates.

'Furthermore, we tested whether FFMQ (Five Facet Mindfulness Questionnaire), Neuroticism, and Resilience had any impact on the self-reported stress reduction effect of the mindfulness condition versus sham. To do so, we incorporated these individual difference measures as covariates in the model to examine their moderating effects on the trajectory of stress reduction over time. Specifically, we extended the latent growth curve model by adding interaction terms between condition and each moderator (FFMQ, Neuroticism, and Resilience), and we estimated separate models for each moderator.'

An additional analysis was also planned to assess demand characteristics:

'Finally, the Perceived Awareness of Research Hypothesis (PARH) scale was evaluated against its midpoint (4.0) using a one-sample t-test to assess demand characteristics [29].'

X26) REB/IRB Approval and Ethical Considerations [recommended as subheading under "Methods"] (not a CONSORT item)

X26-i) Comment on ethics committee approval

	1	2	3	4	5	
subitem not at all important	○	○	●	○	○	essential

Cancella selezione

Does your paper address subitem X26-i?

Copy and paste relevant sections from the manuscript (include quotes in quotation marks "like this" to indicate direct quotes from your manuscript), or elaborate on this item by providing additional information not in the ms, or briefly explain why the item is not applicable/relevant for your study

'This study was conducted in accordance with the Declaration of Helsinki and approved by the A*STAR Institutional Review Board on November 26th 2023 (ASTAR IRB Reference: 2024-102). All participants provided informed consent online via a Qualtrics survey. To protect anonymity, all data, except phone numbers (retained for compensation, HRV data collection, and prevention of duplicate submissions), were de-identified. Phone numbers were deleted at the end of data collection to ensure participant privacy.

[From Recruitment and Eligibility Section]
Before proceeding, participants were informed that the screening included questions designed to determine their eligibility and that honest responses were essential. The survey was configured to present each screening question individually and to terminate immediately if any exclusion criterion was met. This approach was adopted to reduce unnecessary exposure to sensitive questions and to maintain participant comfort and confidentiality.

Eligible participants received a Qualtrics link to review the study protocol and consent form. Due to the study's minimal risk (no medication or incidental findings), consent was obtained remotely without a witness. Participants declining consent were excluded.'

x26-ii) Outline informed consent procedures

Outline informed consent procedures e.g., if consent was obtained offline or online (how? Checkbox, etc.?), and what information was provided (see 4a-ii). See [6] for some items to be included in informed consent documents.

	1	2	3	4	5	
subitem not at all important	○	◉	○	○	○	essential

Cancella selezione

Does your paper address subitem X26-ii?

Copy and paste relevant sections from the manuscript (include quotes in quotation marks "like this" to indicate direct quotes from your manuscript), or elaborate on this item by providing additional information not in the ms, or briefly explain why the item is not applicable/relevant for your study

Informed consent was obtained entirely online through a Qualtrics survey. The procedure was as follows:

Initial Information: Before the formal consent process, prospective participants were informed about the nature of the screening process: 'Before proceeding, participants were informed that the screening included questions designed to determine their eligibility and that honest responses were essential... This approach was adopted to reduce unnecessary exposure to sensitive questions and to maintain participant comfort and confidentiality.'

Provision of Consent Documents: After passing the initial screening, participants were formally presented with the consent materials: 'Eligible participants received a Qualtrics link to review the study protocol and consent form.'

Method of Consent: Consent was provided electronically within the Qualtrics survey. The manuscript notes: 'All participants provided informed consent online via a Qualtrics survey.' and 'Participants declining consent were excluded.' While not explicitly stated, this typically involves participants ticking a checkbox to indicate their agreement after reviewing the materials.

Ethical Considerations for Consent: The process was designed for a minimal-risk remote study: 'Due to the study's minimal risk (no medication or incidental findings), consent was obtained remotely without a witness.'

X26-iii) Safety and security procedures

Safety and security procedures, incl. privacy considerations, and any steps taken to reduce the likelihood or detection of harm (e.g., education and training, availability of a hotline)

	1	2	3	4	5	
subitem not at all important	○	○	◉	○	○	essential

Cancella selezione

Does your paper address subitem X26-iii?

Copy and paste relevant sections from the manuscript (include quotes in quotation marks "like this" to indicate direct quotes from your manuscript), or elaborate on this item by providing additional information not in the ms, or briefly explain why the item is not applicable/relevant for your study

Participant Safety and Comfort during Screening:
"Participants were recruited via an online screening form that assessed eligibility based on several predefined criteria to ensure the safety of participants and the integrity of the research... Before proceeding, participants were informed that the screening included questions designed to determine their eligibility and that honest responses were essential. The survey was configured to present each screening question individually and to terminate immediately if any exclusion criterion was met. This approach was adopted to reduce unnecessary exposure to sensitive questions and to maintain participant comfort and confidentiality."

Exclusion Criteria to Prevent Harm:
The study excluded individuals with specific risk factors to ensure their safety: "Participants were also required to have no history of mental illness, no diagnosed major neurological or psychiatric condition in the past six months, and no current or recent (within the past week) use of psychoactive medications, including antidepressants, anxiolytics, hypnotics, or stimulants."

Data Privacy and Anonymity:
"To protect anonymity, all data, except phone numbers (retained for compensation, HRV data collection, and prevention of duplicate submissions), were de-identified. Phone numbers were deleted at the end of data collection to ensure participant privacy."

No other safety procedures, such as a hotline or specific training, were mentioned as the study was deemed minimal risk.

RESULTS

13a) For each group, the numbers of participants who were randomly assigned, received intended treatment, and were analysed for the primary outcome

NPT: The number of care providers or centers performing the intervention in each group and the number of patients treated by each care provider in each center

Does your paper address CONSORT subitem 13a? *

Copy and paste relevant sections from the manuscript (include quotes in quotation marks "like this" to indicate direct quotes from your manuscript), or elaborate on this item by providing additional information not in the ms, or briefly explain why the item is not applicable/relevant for your study

"A total of 60 participants were randomized, with 30 assigned to the mindfulness intervention and 30 to the sham control condition. All 60 participants received the intended treatment, although one participant discontinued after Day 2 of the 3-day procedure. In accordance with the intention-to-treat principle, all 60 participants were included in the final analysis for the primary outcomes."

13b) For each group, losses and exclusions after randomisation, together with reasons

Does your paper address CONSORT subitem 13b? (NOTE: Preferably, this is shown in a CONSORT flow diagram) *

Copy and paste relevant sections from the manuscript (include quotes in quotation marks "like this" to indicate direct quotes from your manuscript), or elaborate on this item by providing additional information not in the ms, or briefly explain why the item is not applicable/relevant for your study

There were no exclusions after randomization. One participant was lost to follow-up after discontinuing the experiment at the end of Day 2 of the 3-day procedure. The remaining 59 participants completed the full study. The reason for discontinuation was not reported.

13b-i) Attrition diagram

Strongly recommended: An attrition diagram (e.g., proportion of participants still logging in or using the intervention/comparator in each group plotted over time, similar to a survival curve) or other figures or tables demonstrating usage/dose/engagement.

	1	2	3	4	5	
subitem not at all important	○	◉	○	○	○	essential

Cancella selezione

Does your paper address subitem 13b-i?

Copy and paste relevant sections from the manuscript or cite the figure number if applicable (include quotes in quotation marks "like this" to indicate direct quotes from your manuscript), or elaborate on this item by providing additional information not in the ms, or briefly explain why the item is not applicable/relevant for your study

The manuscript reports high-level usage and engagement metrics, including a 98.3% completion rate for all study sessions. However, it does not currently include a figure plotting engagement.

14a) Dates defining the periods of recruitment and follow-up

Does your paper address CONSORT subitem 14a? *

Copy and paste relevant sections from the manuscript (include quotes in quotation marks "like this" to indicate direct quotes from your manuscript), or elaborate on this item by providing additional information not in the ms, or briefly explain why the item is not applicable/relevant for your study

'Participant recruitment began on February 12th 2025 and was completed on March 19th 2025.'

14a-i) Indicate if critical “secular events” fell into the study period

Indicate if critical “secular events” fell into the study period, e.g., significant changes in Internet resources available or “changes in computer hardware or Internet delivery resources”

	1	2	3	4	5	
subitem not at all important	◉	○	○	○	○	essential

Cancella selezione

Does your paper address subitem 14a-i?

Copy and paste relevant sections from the manuscript (include quotes in quotation marks "like this" to indicate direct quotes from your manuscript), or elaborate on this item by providing additional information not in the ms, or briefly explain why the item is not applicable/relevant for your study

No critical “secular events” fell into the study period

14b) Why the trial ended or was stopped (early)

Does your paper address CONSORT subitem 14b? *

Copy and paste relevant sections from the manuscript (include quotes in quotation marks "like this" to indicate direct quotes from your manuscript), or elaborate on this item by providing additional information not in the ms, or briefly explain why the item is not applicable/relevant for your study

Our trial ended because we fulfilled our recruitment criteria.

15) A table showing baseline demographic and clinical characteristics for each group

NPT: When applicable, a description of care providers (case volume, qualification, expertise, etc.) and centers (volume) in each group

Does your paper address CONSORT subitem 15? *

Copy and paste relevant sections from the manuscript (include quotes in quotation marks "like this" to indicate direct quotes from your manuscript), or elaborate on this item by providing additional information not in the ms, or briefly explain why the item is not applicable/relevant for your study

"Baseline demographic and psychological characteristics for each group are presented in Table 1. The final sample comprised 60 participants (M = 33.93 years, SD = 12.18, range = 21-70) with balanced gender representation (50% female, 46.7% male, 3.3% preferred not to disclose). The ethnic composition was 93.3% Chinese, 5% Indian, and 1.7% Malay.

Random allocation successfully produced equal group sizes (n = 30 per condition). As shown in Table 1, the mindfulness group and sham control group showed no statistically significant differences in baseline characteristics (all Ps > .05), suggesting successful randomization."

15-i) Report demographics associated with digital divide issues

In ehealth trials it is particularly important to report demographics associated with digital divide issues, such as age, education, gender, social-economic status, computer/Internet/ehealth literacy of the participants, if known.

	1	2	3	4	5	
subitem not at all important	○	●	○	○	○	essential

Cancella selezione

Does your paper address subitem 15-i? *

Copy and paste relevant sections from the manuscript (include quotes in quotation marks "like this" to indicate direct quotes from your manuscript), or elaborate on this item by providing additional information not in the ms, or briefly explain why the item is not applicable/relevant for your study

The manuscript reports on several demographics relevant to the digital divide:

Age: "The final sample comprised 60 participants (M = 33.93 years, SD = 12.18, range = 21-70)."
Gender: "...with balanced gender representation (50% female, 46.7% male, 3.3% preferred not to disclose)."
Ethnicity: "Ethnic composition reflected the regional demographics, with 93.3% Chinese, 5% Indian, and 1.7% Malay participants."

Computer and internet literacy was not formally measured as a demographic variable:

Computer/Internet Literacy (as an inclusion criterion): "Given that the study procedures were conducted online, participants were also required to have access to the internet and sufficient computer literacy to complete the screening and study tasks." Indirect data on this was collected via the usability questionnaire, which found "Ease of access/navigation (M = 4.21, SD = 0.66)" and "Technical usability... (M = 4.02, SD = 0.64)."

Information on participant education and socio-economic status was also not reported.

16) For each group, number of participants (denominator) included in each analysis and whether the analysis was by original assigned groups

16-i) Report multiple “denominators” and provide definitions

Report multiple “denominators” and provide definitions: Report N’s (and effect sizes) “across a range of study participation [and use] thresholds” [1], e.g., N exposed, N consented, N used more than x times, N used more than y weeks, N participants “used” the intervention/comparator at specific pre-defined time points of interest (in absolute and relative numbers per group). Always clearly define “use” of the intervention.

	1	2	3	4	5	
subitem not at all important	○	○	○	◉	○	essential

Cancella selezione

Does your paper address subitem 16-i? *

Copy and paste relevant sections from the manuscript (include quotes in quotation marks "like this" to indicate direct quotes from your manuscript), or elaborate on this item by providing additional information not in the ms, or briefly explain why the item is not applicable/relevant for your study

The study reports on several denominators reflecting different levels of participation and engagement:

Initial Interest/Screened: A total of 87 participants expressed interest and completed the pre-screening survey.
Randomized: 60 participants were randomized into the two conditions (n=30 per group). This serves as the primary denominator for the intention-to-treat analyses.

Full Protocol Completion ("Use"): "Use" of the intervention was defined as completing the full 3-day protocol. 59 of 60 participants (98.3%) met this definition. One participant partially completed the protocol, discontinuing after Day 2.

Data Submission Engagement: A secondary metric of engagement was the submission of HRV data files. 57 of 60 participants (95%) successfully completed this task.

16-ii) Primary analysis should be intent-to-treat

Primary analysis should be intent-to-treat, secondary analyses could include comparing only “users”, with the appropriate caveats that this is no longer a randomized sample (see 18-i).

	1	2	3	4	5	
subitem not at all important	○	◉	○	○	○	essential

Cancella selezione

Does your paper address subitem 16-ii?

Copy and paste relevant sections from the manuscript (include quotes in quotation marks "like this" to indicate direct quotes from your manuscript), or elaborate on this item by providing additional information not in the ms, or briefly explain why the item is not applicable/relevant for your study

The primary analyses were conducted on an intention-to-treat (ITT) basis. All participants were analyzed according to the group to which they were originally randomized. As stated in the manuscript

'All 60 participants were included in the final analysis. One participant discontinued the experiment after the end of Day 2, but was retained in the analysis with available data.'"

17a) For each primary and secondary outcome, results for each group, and the estimated effect size and its precision (such as 95% confidence interval)

Does your paper address CONSORT subitem 17a? *

Copy and paste relevant sections from the manuscript (include quotes in quotation marks "like this" to indicate direct quotes from your manuscript), or elaborate on this item by providing additional information not in the ms, or briefly explain why the item is not applicable/relevant for your study

The manuscript reports results for each primary and secondary outcome, using Bayes Factors (BF_{10}) as the primary measure of evidence (effect size) and providing confidence intervals where traditional frequentist tests were used. A $BF_{10} < 1$ provides evidence for the null hypothesis (no difference), while a $BF_{10} > 1$ provides evidence for the alternative hypothesis.

Primary Outcomes:
Feasibility and Acceptability: These were assessed descriptively rather than with effect sizes.
Feasibility: The study achieved a 69% recruitment yield, 98.3% retention, and 95% HRV data submission rate.
Acceptability: Overall usability was favorable (M = 4.17, SD = 0.53).
Self-Reported Stress (STAI-6):
Group Comparison: "Bayesian analyses revealed strong evidence for the null hypothesis for self-reported stress (STAI-6), indicating no differential improvement between conditions ($BF_{10} = 0.03$)."
Overall Effect of Time: A significant reduction in anxiety was observed across both groups over time ($BF_{10} = 3.01 \times 10^6$).

Secondary Outcomes:
Heart Rate Variability (RMSSD):
Group Comparison: "For heart rate variability (RMSSD), the results showed anecdotal evidence for the null hypothesis ($BF_{10} = 0.20$)."
Ecological Momentary Assessments (EMAs):
Group Comparison: "All analyses yielded inconclusive Bayes factors ($BF_{10} < 1$), indicating no statistically detectable differences between the mindfulness and sham meditation groups."
Other Analyses with Effect Sizes and Precision:

Demand Characteristics (PARH): The analysis showed no significant effect, with the precision captured by the confidence interval. "The analysis indicated that the mean PARH score (M = 4.10 SD=1.88) was not significantly different from the scale midpoint, $t(56)=0.40$, $P=.689$, 95% CI [3.60,4.60]."
Moderator Analyses: The effect size for moderation was negligible, with strong evidence for the null models for trait mindfulness ($BF_{10} = 0.009$), neuroticism ($BF_{10} = 0.116$), and resilience ($BF_{10} = 0.005$).
Baseline Group Differences: Cohen's d was reported for baseline continuous measures, showing small and non-significant differences, for example: IPIP (d = -0.24), RS (d = 0.11), and FFMQ (d = 0.30).

17a-i) Presentation of process outcomes such as metrics of use and intensity of use

In addition to primary/secondary (clinical) outcomes, the presentation of process outcomes such as metrics of use and intensity of use (dose, exposure) and their operational definitions is critical. This does not only refer to metrics of attrition (13-b) (often a binary variable), but also to more continuous exposure metrics such as “average session length”. These must be accompanied by a technical description how a metric like a “session” is defined (e.g., timeout after idle time) [1] (report under item 6a).

	1	2	3	4	5	
subitem not at all important	○	○	○	◉	○	essential

Cancella selezione

Does your paper address subitem 17a-i?

Copy and paste relevant sections from the manuscript (include quotes in quotation marks "like this" to indicate direct quotes from your manuscript), or elaborate on this item by providing additional information not in the ms, or briefly explain why the item is not applicable/relevant for your study

The manuscript reports results for each primary and secondary outcome, using Bayes Factors (BF_{10}) as the primary measure of evidence and effect size. A $BF_{10} < 1$ provides evidence for the null hypothesis, while a $BF_{10} > 1$ provides evidence for the alternative hypothesis.

Primary Outcomes:

Feasibility and Acceptability: These were assessed descriptively.

Feasibility: "successfully recruiting 60 participants from 87 screened (69% yield) with near-perfect retention (59/60 completed all sessions; 98.3%)." HRV data was submitted by 95% of participants.
Acceptability: "Quantitative usability ratings... indicated favourable evaluations overall (M = 4.17, SD = 0.53)."

Self-Reported Stress (STAI-6):
Group Comparison: "Bayesian analyses revealed strong evidence for the null hypothesis for self-reported stress (STAI-6), indicating no differential improvement between conditions ($BF_{10} = 0.03$)."
Overall Effect of Time: Both groups showed significant pre-to-post reductions in anxiety across timepoints, irrespective of condition ($BF_{10} = 3.01 \times 10^6$).
Secondary Outcomes:
Heart Rate Variability (RMSSD):
Group Comparison: "For heart rate variability (RMSSD), the results showed anecdotal evidence for the null hypothesis ($BF_{10} = 0.20$)."
Correlation with Stress: A moderate negative correlation was found between RMSSD and self-reported stress ($r = -0.28$, $P < .001$).
Ecological Momentary Assessments (EMAs): "All analyses yielded inconclusive Bayes factors ($BF_{10} < 1$), indicating no statistically detectable differences between the mindfulness and sham meditation groups."
Additional Analyses:
Demand Characteristics (PARH): No effect was found. "The analysis indicated that the mean PARH score (M = 4.10 SD=1.88) was not significantly different from the scale midpoint, $t(56)=0.40$, $P = .689$, 95% CI [3.60,4.60]."
Expectancy: The mindfulness group reported higher expectancy. "The mindfulness condition yielded higher expectancy scores (M = 6.69, SD = 2.19) compared to the sham condition (M = 5.13, SD = 2.40)." However, adjusting for this did not change the primary outcome (BF_{10} remained 0.03).
Credibility: There was a non-significant trend for the mindfulness group to better identify their condition. "$\chi^2(1) = 3.18$, $P = .074$."
Moderator Analyses (FFMQ, Neuroticism, Resilience): No moderation effects were found. Analyses showed strong evidence for the null models for all three traits: FFMQ ($BF_{10} = 0.009$), neuroticism ($BF_{10} = 0.116$), and resilience ($BF_{10} = 0.005$).

17b) For binary outcomes, presentation of both absolute and relative effect sizes is recommended

Does your paper address CONSORT subitem 17b? *

Copy and paste relevant sections from the manuscript (include quotes in quotation marks "like this" to indicate direct quotes from your manuscript), or elaborate on this item by providing additional information not in the ms, or briefly explain why the item is not applicable/relevant for your study

This item is not applicable to the primary or secondary outcome analyses reported in your manuscript.
All of the main outcome variables you analyzed (STAI-6, RMSSD, EMAs, feasibility percentages) were treated as continuous or count data, not binary outcomes. Therefore, the recommendation to present both absolute and relative effect sizes for binary outcomes does not apply to your statistical approach.

18) Results of any other analyses performed, including subgroup analyses and adjusted analyses, distinguishing pre-specified from exploratory

Does your paper address CONSORT subitem 18? *

Copy and paste relevant sections from the manuscript (include quotes in quotation marks "like this" to indicate direct quotes from your manuscript), or elaborate on this item by providing additional information not in the ms, or briefly explain why the item is not applicable/relevant for your study

The manuscript clearly distinguishes between pre-specified and exploratory analyses and reports the results for each.
Pre-specified Additional Analyses:
The following analyses were pre-specified in the Methods and their results are reported:
Moderator Analysis: "Examinations of baseline individual differences revealed no evidence for moderation effects on self-reported stress. Bayes factor analyses indicated strong evidence in favour of the reduced models (i.e., without moderation terms) over the full models including interactions for trait mindfulness (FFMQ; BF_{10} = 0.009), neuroticism (BF_{10} = 0.116), and resilience (BF_{10} = 0.005)."
Demand Characteristics Analysis: "The analysis indicated that the mean PARH score (M = 4.10 SD=1.88) was not significantly different from the scale midpoint, t(56)=0.40, P =.689, 95% CI [3.60,4.60]. This suggests that, on average, participants did not report a clear awareness of the research hypotheses."
Expectancy and Credibility Analyses: The manuscript reports that the mindfulness group had higher expectancy scores. An adjusted analysis was performed: "We adjusted our primary Bayesian mixed-effects model for STAI by including baseline expectancy scores as a covariate. The results confirmed our original conclusion: after adjusting for expectancy, the analysis continued to show strong evidence for the null hypothesis... yielding a Bayes Factor of BF10 = 0.03." The credibility analysis showed a non-significant trend ($\chi^2(1)$ = 3.18, P = .074).

Exploratory Analyses:
The manuscript does not label any analyses as "exploratory" in the results section, however, the section titled "Difference Pre to Post" can be considered an exploratory or post-hoc analysis designed to understand the main effect of time, given the null finding for the primary group comparison.
Analysis of Temporal Changes (Pre- to Post-Intervention): "Bayesian latent growth curve modelling provided statistically significant evidence for reductions in self-reported stress measured via STAI-6 over time, regardless of experimental condition... This comparison yielded a Bayes Factor of 3.01×10^6 (BF_{10}) strongly favouring the temporal model over the intercept-only null model."

18-i) Subgroup analysis of comparing only users

A subgroup analysis of comparing only users is not uncommon in ehealth trials, but if done, it must be stressed that this is a self-selected sample and no longer an unbiased sample from a randomized trial (see 16-iii).

	1	2	3	4	5	
subitem not at all important	(x)	( )	( )	( )	( )	essential

Cancella selezione

Does your paper address subitem 18-i?

Copy and paste relevant sections from the manuscript (include quotes in quotation marks "like this" to indicate direct quotes from your manuscript), or elaborate on this item by providing additional information not in the ms, or briefly explain why the item is not applicable/relevant for your study

This specific subgroup analysis was not performed. The primary analysis was conducted on an intention-to-treat basis, including all 60 randomized participants regardless of their level of completion, as described in the statistical methods.

19) All important harms or unintended effects in each group

(for specific guidance see CONSORT for harms)

Does your paper address CONSORT subitem 19? *

Copy and paste relevant sections from the manuscript (include quotes in quotation marks "like this" to indicate direct quotes from your manuscript), or elaborate on this item by providing additional information not in the ms, or briefly explain why the item is not applicable/relevant for your study

No serious or unexpected adverse effects were reported during the trial.

19-i) Include privacy breaches, technical problems

Include privacy breaches, technical problems. This does not only include physical “harm” to participants, but also incidents such as perceived or real privacy breaches [1], technical problems, and other unexpected/unintended incidents. “Unintended effects” also includes unintended positive effects [2].

	1	2	3	4	5	
subitem not at all important	○	○	●	○	○	essential

Cancella selezione

Does your paper address subitem 19-i?

Copy and paste relevant sections from the manuscript (include quotes in quotation marks "like this" to indicate direct quotes from your manuscript), or elaborate on this item by providing additional information not in the ms, or briefly explain why the item is not applicable/relevant for your study

No privacy breaches or technical problems were encountered during the trial.

19-ii) Include qualitative feedback from participants or observations from staff/researchers

Include qualitative feedback from participants or observations from staff/researchers, if available, on strengths and shortcomings of the application, especially if they point to unintended/unexpected effects or uses. This includes (if available) reasons for why people did or did not use the application as intended by the developers.

	1	2	3	4	5	
subitem not at all important	○	○	●	○	○	essential

Cancella selezione

Does your paper address subitem 19-ii?

Copy and paste relevant sections from the manuscript (include quotes in quotation marks "like this" to indicate direct quotes from your manuscript), or elaborate on this item by providing additional information not in the ms, or briefly explain why the item is not applicable/relevant for your study

The manuscript provides qualitative feedback from participants, which highlighted several strengths and shortcomings of the intervention and study design.

Shortcomings and Challenges Identified by Participants:
The most commonly reported challenges included:
Technical Issues: 23.5% of participants reported problems with the HRV measurement app, including specific unintended effects like phone overheating and one participant stating the "flashlight burned my finger."
Adherence Difficulties: 21.6% found it difficult to maintain a consistent schedule and remember the daily tasks, with one noting the challenge of "sticking to the same time every day" without reminders.
Engagement Issues: 17.6% reported difficulty focusing during the meditation sessions.
Usability of Instructions: 13.7% found the instructions fragmented, with one describing it as, "Jumping back and forth between these 3 modes was quite distracting."
Strengths Identified by Participants:
Strengths were reflected in the quantitative usability ratings, which showed "favourable evaluations overall (M = 4.17, SD = 0.53), with consistently positive scores across subscales: Ease of access/navigation (M = 4.21, SD = 0.66), clarity of instructions (M = 4.18, SD = 0.69), comfort/engagement (M = 4.27, SD = 0.57), and procedural acceptability (M = 4.14)."
Participant Suggestions for Improvement:
When asked for suggestions, participants recommended "streamlining instructions (15.7%, n = 8) and implementing daily reminders (7.8%, n = 4)."

DISCUSSION

22) Interpretation consistent with results, balancing benefits and harms, and considering other relevant evidence

NPT: In addition, take into account the choice of the comparator, lack of or partial blinding, and unequal expertise of care providers or centers in each group

22-i) Restate study questions and summarize the answers suggested by the data, starting with primary outcomes and process outcomes (use)

Restate study questions and summarize the answers suggested by the data, starting with primary outcomes and process outcomes (use).

	1	2	3	4	5	
subitem not at all important	○	○	○	○	◉	essential

Cancella selezione

Does your paper address subitem 22-i? *

Copy and paste relevant sections from the manuscript (include quotes in quotation marks "like this" to indicate direct quotes from your manuscript), or elaborate on this item by providing additional information not in the ms, or briefly explain why the item is not applicable/relevant for your study

"The primary aim of this pilot trial was to evaluate the feasibility and acceptability of a fully decentralized Self-Administered Mindfulness (SAM) intervention that incorporated a structurally matched sham control and smartphone-based HRV monitoring.

The findings strongly support the primary outcomes related to feasibility and process outcomes (use):

Feasibility: 'This study demonstrated strong feasibility for decentralized delivery, with a participant retention rate of 98.3%, which surpasses established benchmarks. Protocol adherence was high overall; participants independently completed all study procedures, including HRV recordings, online surveys, and audio-guided sessions with minimal supervision.' However, a key challenge was the '30.2% data exclusion rate' for HRV recordings due to signal quality issues.

Acceptability & Use: 'The study demonstrated strong evidence of acceptability across multiple evaluation approaches. Participants generally found the study materials, delivery platforms, and procedures accessible and manageable, as reflected in both quantitative ratings and qualitative feedback.'

For the primary clinical outcome of self-reported stress, the study found no difference between the intervention and the comparator:

Stress (STAI-6): 'Bayesian analyses revealed strong evidence for the null hypothesis for self-reported stress (STAI-6), indicating no differential improvement between conditions (BF_{10} = 0.03).' However, 'both groups showed significant pre-to-post reductions in anxiety (BF_{10} = 3.01 × 10^6).'"

22-ii) Highlight unanswered new questions, suggest future research

Highlight unanswered new questions, suggest future research.

	1	2	3	4	5	
subitem not at all important	○	○	○	◉	○	essential

Cancella selezione

Does your paper address subitem 22-ii?

Copy and paste relevant sections from the manuscript (include quotes in quotation marks "like this" to indicate direct quotes from your manuscript), or elaborate on this item by providing additional information not in the ms, or briefly explain why the item is not applicable/relevant for your study

This information is clearly detailed throughout your Discussion and is summarized in the Conclusion. The manuscript raises several new questions and provides a roadmap for future research.

➡️ What to paste:

The manuscript highlights several unanswered questions and provides specific suggestions for future research:

1. Regarding Intervention Efficacy and Duration:
The study questions whether brief interventions can isolate mindfulness-specific effects and suggests future research should focus on longer durations and follow-up.

"Whether longer or more intensive interventions would produce stronger alignment between physiological and self-reported outcomes remains an open and important question."

"Future work should extend these findings to larger, more diverse samples with longer-term follow-up..."

2. Regarding Sham Control Design:
The study raises questions about the perceptual equivalence of the sham control and calls for more work in this area.

"...future research must continue to develop more sophisticated controls to fully disentangle the specific versus non-specific drivers of stress reduction in remote interventions."

"This pilot study identified the need for further sham refinement and validation prior to full-scale intervention launch."

3. Regarding Technical and Logistical Challenges of Decentralized Trials:
The manuscript identifies the need to improve remote physiological data collection and the overall user experience.

"The observed challenges underscore the need for enhanced onboarding materials, such as step-by-step instructional videos or in-app quality checks, especially to mitigate data loss on the Android platform [38]."

The discussion also advocates for "open repositories of usability feedback and de-identified adherence data resources that could help standardize best practices across studies" and suggests "improved app functionality (e.g., features to prevent overheating during PPG recordings)."

4. Regarding Generalizability:
The limitations section points to the need for research in broader populations.

"Cultural differences in stress expression and coping may limit applicability to broader populations." Future studies should include more diverse samples beyond the specific demographic reported.

20) Trial limitations, addressing sources of potential bias, imprecision, and, if relevant, multiplicity of analyses

20-i) Typical limitations in ehealth trials

Typical limitations in ehealth trials: Participants in ehealth trials are rarely blinded. Ehealth trials often look at a multiplicity of outcomes, increasing risk for a Type I error. Discuss biases due to non-use of the intervention/usability issues, biases through informed consent procedures, unexpected events.

	1	2	3	4	5	
subitem not at all important	○	○	○	○	◉	essential

Cancella selezione

Does your paper address subitem 20-i? *

Copy and paste relevant sections from the manuscript (include quotes in quotation marks "like this" to indicate direct quotes from your manuscript), or elaborate on this item by providing additional information not in the ms, or briefly explain why the item is not applicable/relevant for your study

Several limitations temper the interpretation of our findings.
Potential for Imprecision (Power): "First, while our sample size (n = 60) was adequate for assessing feasibility and acceptability [42], it was underpowered to detect subtle between-group differences or moderation effects."
Limited Intervention Dose: "Second the total guided practice time was notably brief, amounting to only 30 minutes across three sessions. This minimal dosage, while practical for a feasibility trial, may only be sufficient to capture immediate, short-term effects rather than inducing substantial or lasting changes in well-being..."
Lack of Follow-Up: "...and the lack of follow-up assessments precludes evaluation of the intervention's long-term effects."
Sources of Potential Bias (Measurement): "Fourth, in line with the study's aim to test a flexible and ecologically valid decentralized intervention, we did not mandate a specific time of day for the weekly HRV assessments... unmeasured circadian variations in heart rate variability are a limitation of this study and may have introduced additional noise into the physiological data."
Constrained Generalizability: "Third, our study has constrained generalizability [43]. The findings are most applicable to: 1) English-fluent adults (>21 years) in Singapore, 2) Non-meditators (≥6 months) without mental health histories or medication use, and 3) Predominantly Chinese Singaporean participants (93.3%)."

21) Generalisability (external validity, applicability) of the trial findings

NPT: External validity of the trial findings according to the intervention, comparators, patients, and care providers or centers involved in the trial

21-i) Generalizability to other populations

Generalizability to other populations: In particular, discuss generalizability to a general Internet population, outside of a RCT setting, and general patient population, including applicability of the study results for other organizations

	1	2	3	4	5	
subitem not at all important	○	○	○	◉	○	essential

Cancella selezione

Does your paper address subitem 21-i?

Copy and paste relevant sections from the manuscript (include quotes in quotation marks "like this" to indicate direct quotes from your manuscript), or elaborate on this item by providing additional information not in the ms, or briefly explain why the item is not applicable/relevant for your study

'Limitations and Constraints on Generality

Several limitations temper the interpretation of our findings. First, while our sample size (n = 60) was adequate for assessing feasibility and acceptability [42], it was underpowered to detect subtle between-group differences or moderation effects. Second the total guided practice time was notably brief, amounting to only 30 minutes across three sessions. This minimal dosage, while practical for a feasibility trial, may only be sufficient to capture immediate, short-term effects rather than inducing substantial or lasting changes in well-being and the lack of follow-up assessments precludes evaluation of the intervention's long-term effects. Third, our study has constrained generalizability [43]. The findings are most applicable to: 1) English-fluent adults (>21 years) in Singapore, 2) Non-meditators (≥6 months) without mental health histories or medication use, and 3) Predominantly Chinese Singaporean participants (93.3%). Cultural differences in stress expression and coping may limit applicability to broader populations. Fourth, in line with the study's aim to test a flexible and ecologically valid decentralized intervention, we did not mandate a specific time of day for the weekly HRV assessments. While participants were encouraged to complete these sessions at a consistent time to minimize circadian effects, we could not enforce or verify their adherence. Therefore, unmeasured circadian variations in heart rate variability are a limitation of this study and may have introduced additional noise into the physiological data.
'

21-ii) Discuss if there were elements in the RCT that would be different in a routine application setting

Discuss if there were elements in the RCT that would be different in a routine application setting (e.g., prompts/reminders, more human involvement, training sessions or other co-interventions) and what impact the omission of these elements could have on use, adoption, or outcomes if the intervention is applied outside of a RCT setting.

	1	2	3	4	5	
subitem not at all important	○	○	○	○	○	essential

Does your paper address subitem 21-ii?

Copy and paste relevant sections from the manuscript (include quotes in quotation marks "like this" to indicate direct quotes from your manuscript), or elaborate on this item by providing additional information not in the ms, or briefly explain why the item is not applicable/relevant for your study

Yes, the trial identified several elements that were absent in the RCT that would be beneficial in a routine application setting. The omission of these elements was noted by participants and impacted their experience.

1. Prompts/Reminders:
The RCT did not use reminders, and participants identified this as a key challenge. In a routine setting, adding reminders would be crucial for adherence.

Participant Feedback: A key challenge was "maintaining consistent timing and remembering to complete tasks (21.6%, n = 11) without reminders."
Recommendation for Routine Application: The study recommends to "Send automated reminders (SMS/email)" to "Lower missed assessments without staff effort" (Table 3).
2. Training Sessions (Onboarding):
While the RCT provided written instructions, feedback indicated this was insufficient, especially for technical tasks. A routine application would require enhanced training.

Participant Feedback: Participants noted "Technical issues with the HRV measurement app (23.5%, n = 12)."
Recommendation for Routine Application: The study recommends to "Use video tutorials for technical tasks" to "Minimize technical errors, enhances adherence, and promotes participant autonomy" (Table 3).
3. Human Involvement and Platform Integration:
The RCT required participants to navigate multiple platforms. A routine application would benefit from less "human involvement" in the sense of self-navigation and more integration.

Participant Feedback: A challenge was "navigating fragmented instructions (13.7%, n = 7) ('Jumping back and forth between these 3 modes was quite distracting')."
Recommendation for Routine Application: The study recommends to "Integrate all tools into one user-friendly system" to "Prevent confusion and Streamline user experience" (Table 3).
The omission of these elements in the RCT directly led to participant-reported challenges in adherence, usability, and cognitive load. Implementing these changes in a routine application would likely improve adoption, sustained use, and potentially the quality of data collected.

OTHER INFORMATION

23) Registration number and name of trial registry

Does your paper address CONSORT subitem 23? *

Copy and paste relevant sections from the manuscript (include quotes in quotation marks "like this" to indicate direct quotes from your manuscript), or elaborate on this item by providing additional information not in the ms, or briefly explain why the item is not applicable/relevant for your study

yes - This study was preregistered on ClinicalTrials.gov (Registration ID: NCT06765889).

24) Where the full trial protocol can be accessed, if available

Does your paper address CONSORT subitem 24? *

Cite a Multimedia Appendix, other reference, or copy and paste relevant sections from the manuscript (include quotes in quotation marks "like this" to indicate direct quotes from your manuscript), or elaborate on this item by providing additional information not in the ms, or briefly explain why the item is not applicable/relevant for your study

This study was preregistered on ClinicalTrials.gov (Registration ID: NCT06765889)

25) Sources of funding and other support (such as supply of drugs), role of funders

Does your paper address CONSORT subitem 25? *

Copy and paste relevant sections from the manuscript (include quotes in quotation marks "like this" to indicate direct quotes from your manuscript), or elaborate on this item by providing additional information not in the ms, or briefly explain why the item is not applicable/relevant for your study

'This study is funded out of the A*STAR (Agency for Science, Technology and Research) BMRC (Biomedical Research Council) CRF (Central Research Fund) grant of the Principal Investigator.'

X27) Conflicts of Interest (not a CONSORT item)

X27-i) State the relation of the study team towards the system being evaluated

In addition to the usual declaration of interests (financial or otherwise), also state the relation of the study team towards the system being evaluated, i.e., state if the authors/evaluators are distinct from or identical with the developers/sponsors of the intervention.

	1	2	3	4	5	
subitem not at all important	○	○	○	◉	○	essential

Cancella selezione

Does your paper address subitem X27-i?

Copy and paste relevant sections from the manuscript (include quotes in quotation marks "like this" to indicate direct quotes from your manuscript), or elaborate on this item by providing additional information not in the ms, or briefly explain why the item is not applicable/relevant for your study

The authors have no competing interests to declare

About the CONSORT EHEALTH checklist

As a result of using this checklist, did you make changes in your manuscript? *

- ○ yes, major changes
- ◉ yes, minor changes
- ○ no

What were the most important changes you made as a result of using this checklist?

Using this checklist was instrumental in improving the manuscript's rigor and transparency. I added critical information that was previously omitted, in the Title, Abstract, Methods and Results sections. Furthermore, I have now included the specific recruitment dates.

How much time did you spend on going through the checklist INCLUDING making changes in your manuscript *

I think it was 3 to 4 hours.

As a result of using this checklist, do you think your manuscript has improved? *

- (x) yes
- ( ) no
- ( ) Altro:

Would you like to become involved in the CONSORT EHEALTH group?

This would involve for example becoming involved in participating in a workshop and writing an "Explanation and Elaboration" document

- ( ) yes
- (x) no
- ( ) Altro:

Cancella selezione

Any other comments or questions on CONSORT EHEALTH

no other comments on CONSORT EHEALTH.

STOP - Save this form as PDF before you click submit

To generate a record that you filled in this form, we recommend to generate a PDF of this page (on a Mac, simply select "print" and then select "print as PDF") before you submit it.

When you submit your (revised) paper to JMIR, please upload the PDF as supplementary file.

Don't worry if some text in the textboxes is cut off, as we still have the complete information in our database. Thank you!

Final step: Click submit !

Click submit so we have your answers in our database!

Invia

Cancella modulo

Non inviare mai le password tramite Moduli Google.

Questi contenuti non sono creati né avallati da Google. - Contatta il proprietario del modulo - Termini di servizio - Norme sulla privacy

Questo modulo sembra sospetto? Segnala

Google Moduli
